# Integrative analysis of genome-wide gene copy number changes and gene expression in non-small cell lung cancer

**DOI:** 10.1371/journal.pone.0187246

**Published:** 2017-11-07

**Authors:** Verena Jabs, Karolina Edlund, Helena König, Marianna Grinberg, Katrin Madjar, Jörg Rahnenführer, Simon Ekman, Michael Bergkvist, Lars Holmberg, Katja Ickstadt, Johan Botling, Jan G. Hengstler, Patrick Micke

**Affiliations:** 1 Faculty of Statistics, TU Dortmund University, Dortmund, Germany; 2 Leibniz Research Centre for Working Environment and Human Factors (IfADo) at Dortmund University, Dortmund, Germany; 3 Department of Oncology, Karolinska University Hospital, Stockholm, Sweden; 4 Dept. of Oncology, Gävle Hospital, Gävle, Sweden; 5 Regional Cancer Center Uppsala-Örebro, Uppsala, Sweden; 6 King’s College London, Faculty of Life Sciences and Medicine, Division of Cancer Studies, London, United Kingdom; 7 Dept. of Immunology, Genetics and Pathology, Uppsala University, Uppsala, Sweden; Roswell Park Cancer Institute, UNITED STATES

## Abstract

Non-small cell lung cancer (NSCLC) represents a genomically unstable cancer type with extensive copy number aberrations. The relationship of gene copy number alterations and subsequent mRNA levels has only fragmentarily been described. The aim of this study was to conduct a genome-wide analysis of gene copy number gains and corresponding gene expression levels in a clinically well annotated NSCLC patient cohort (n = 190) and their association with survival. While more than half of all analyzed gene copy number-gene expression pairs showed statistically significant correlations (10,296 of 18,756 genes), high correlations, with a correlation coefficient >0.7, were obtained only in a subset of 301 genes (1.6%), including KRAS, EGFR and MDM2. Higher correlation coefficients were associated with higher copy number and expression levels. Strong correlations were frequently based on few tumors with high copy number gains and correspondingly increased mRNA expression. Among the highly correlating genes, GO groups associated with posttranslational protein modifications were particularly frequent, including ubiquitination and neddylation. In a meta-analysis including 1,779 patients we found that survival associated genes were overrepresented among highly correlating genes (61 of the 301 highly correlating genes, FDR adjusted p<0.05). Among them are the chaperone CCT2, the core complex protein NUP107 and the ubiquitination and neddylation associated protein CAND1. In conclusion, in a comprehensive analysis we described a distinct set of highly correlating genes. These genes were found to be overrepresented among survival-associated genes based on gene expression in a large collection of publicly available datasets.

## Introduction

NSCLC represents a morphologically and clinically heterogeneous cancer type, with overall poor prognosis [1]. In small subsets of patients, distinct genomic aberrations have been identified that are now successfully exploited for therapeutic intervention. These clinically relevant molecular events are either activating mutations, e.g. mutations in the receptor tyrosine kinases EGFR, BRAF, or HER2, or fusion genes created by gene rearrangements involving ALK or ROS1 [2–3]. Recently, amplifications of FGFR1 and MET were also identified as cancer driving mechanisms and were tested as potential therapeutic targets in clinical trials [4–6].

NSCLC is characterized by genomic instability, with a high frequency of somatic mutations and extensive gene copy number variations in individual lung cancer genomes [7–8]. Genomic alterations can either be focal or include larger regions and entire chromosomal arms [9–10]. For some genes, copy number alterations have been shown to correlate with mRNA expression. A well-known example is HER2 amplification in breast [11–12] and gastric cancer [13], which translates into higher gene and protein expression and define distinct biological subgroups in these cancers. Such relations have also been described in NSCLC [8, 14–19]. However, factors influencing the correlation between gene copy number and gene expression level have not been thoroughly described in NSCLC, neither has it been clarified to which degree genes displaying a high correlation between expression level and copy number are associated overall survival. Therefore, a genome-wide analysis of gene copy number and corresponding gene expression was performed in a clinically well-characterized NSCLC patient cohort.

## Methods

### Patient cohort

Tumor tissue from 190 NSCLC patients was analyzed for both global gene expression levels and genome-wide gene copy numbers using array-based technologies. All analyzed specimens were procured within the infrastructure of an established biobank and stored at -80°C until later use. Selection criteria for study inclusion have been described previously [9, 20]. In brief, tissue specimens from surgically resected non-small cell lung cancer patients, operated 1995–2005 at the Uppsala University Hospital, fulfilling the inclusion criteria of (i) NSCLC histology (adenocarcinoma, squamous cell carcinoma or large cell carcinoma) confirmed by hematoxylin-eosin staining of the frozen tissue sample, (ii) tumor specimen larger than five mm, (iii) tumor cell fraction >50% in analyzed specimen and (iv) RNA integrity (RIN) value >7, as assessed on the Agilent 2100 Bioanalyzer (Agilent Biotechnologies, Palo Alto, USA), were analyzed. Information on clinicopathological parameters and overall survival time (S1 Table) was obtained from the records of the population-based Uppsala-Örebro Regional Lung Cancer Register. The analysis of human tissue specimens and corresponding clinicopathological data was approved by the Uppsala Regional Ethical Review Board (#2006/325) and performed in accordance with Swedish biobank legislation.

### Microarray data preprocessing and analysis

For 190 NSCLC patients, global gene expression (GE) analysis was previously performed using Affymetrix HG U133 Plus 2.0 arrays (GSE37745, [20]). The raw microarray data was normalized by robust multi-array average (RMA) [21–22] using the R package ‘affy’ [23].

Genome-wide single nucleotide polymorphism (SNP) analysis was performed using Affymetrix Gene Chip Human Mapping 250K Nsp I arrays, as previously described [9]. The analysis of SNP array data from 100 of these patients have been published previously (GSE28582 [9]). The data of this study, in total 190 cases, has been deposited under accession number GSE76730. Copy numbers (CN) were estimated using Robust Multichip Analysis (CRMA v2) as implemented in the R package ‘aroma.affymetrix’ [24]. The preprocessing included quantile normalization [23], fitting a log-additive probe-level model [21] for probe summarization, combining the alleles and merging the strands, and fragment length normalization [25]. CN estimates for each of the 261801 SNPs were calculated as the ratio of the preprocessed signal intensity and median across 90 non-cancer HapMap reference samples (The HapMap project, 2003). These non-log scale values were multiplied by two and accordingly, a value of 2 implies equal CN in the cohort to the median of HapMap reference [26]. Circular binary segmentation (CBS) with default parameters was applied to the SNP-wise estimated CNs [27–28]. Calls for normal CN, loss, gain, high gain or homozygous deletion were assigned to each of the 261801 SNPs using a two-level hierarchical mixture model which utilizes the breakpoint information from the CBS [29]. CN frequency plots were created using the R package ‘CGHcall’ [29], plotting the percentage of patients with a particular aberration (CN gain, high gain, CN loss, homozygous deletion) across chromosomal positions.

Modified Manhattan plots based on the function ‘mhtplot’ in the R package ‘gap’ [30] were created to illustrate genome wide segmented CN values. In the Manhattan plots, 95% percentiles of segmented CN values are shown, because often only a relatively small fraction of patients showed aberrant copy numbers, which would not be represented by e.g. the median of segmented CN values. Gene expression was illustrated by plotting the median expression value for each probe set. The simple moving median as well as the 5% and 95% moving quantile of gene expression were calculated from 301 probe sets and added to the GE plot.

### Genome-wide correlation analysis

The GE value for each of the 54675 probe sets included on the Affymetrix HG U133 Plus 2.0 array was assigned to its respective chromosomal position and matched to corresponding segmented CN estimate. The R package ‘hgu133plus2.db’ [31] provided information about chromosome number and start-stop positions for the individual probes that constitute a probe set. Probe sets with missing or ambiguous information of genomic localization were omitted. Probe sets mapping to the Y chromosome were not covered on the Affymetrix Gene Chip Mapping 250K Nsp I array and were also omitted. For each of the remaining 39788 probe sets, the mean chromosomal position was calculated using the minimum and maximum start and stop positions. The 39788 probe sets corresponded to 18756 annotated genes with segmented copy number estimates. To assess the correlation between GE and CN, the externally centered correlation coefficient (ECCC) was calculated, essentially as described by Schäfer et al. [32] using the R package ‘edira’ [33]. For each probe set, ECCC considers the equally directed deviations of CN and GE intensities from the median of an external reference group. The HapMap data set [26] was used as external reference to center the CN data and the median expression value was used to center the GE data. The Wilcoxon signed rank test was used to test for deviations from the reference median for equally directed abnormalities. In contrast to Schäfer et al. [32], linear CN and GE values were included, i.e. the data was not log_2_-transformed.

Modified Manhattan plots (the exact code based on the function ‘mhtplot’ in the ‘gap’ R package [30] is available upon request) were created to illustrate median gene expression and the ECCC. Scatter plots were generated to visualize the relation between gene expression and gene copy number, with linear GE values on the y-axis and matched CN values on the x-axis. Median ECCC of ‘cancer genes’ (1728 probesets corresponding to 533 ‘cancer genes’ defined by the Cancer Gene Census (http://cancer.sanger.ac.uk/cancergenome/projects/census/, accessed 11/2015 [34]) were compared to all other probe sets by the Wilcoxon test and were visualized as box plots. To test whether differences between 'cancer genes' and all other genes were independent from copy number and gene expression a multivariate model was applied, which was stratified for (i) copy number (as a continuous variable using the copy number of all genes) and (ii) gene expression (as a continuous variable using the expression level of all genes). The same method was used to test whether differences in ECCC between 43 lung cancer associated genes were independent from copy number and gene expression.

To further validate the correlations between CN and GE in an independent cohort, Affymetrix SNP 6.0 and RNA-seq data generated by the Cancer Genome Atlas (TCGA) were accessed via the cBioPortal (http://www.cbioportal.org), [35–36]. Data from 520 adenocarcinomas (LUAD; Query: TCGA provisional) and 504 squamous cell cancers (LUSC; Query: TCGA provisional) were analyzed. The data for GE (continuous) and CN (categorized as putative copy-number calls determined using GISTIC 2.0, where -2 = homozygous deletion; -1 = hemizygous deletion; 0 = neutral / no change; 1 = gain; 2 = high level amplification) were downloaded via the cBioPortal and analyzed using Spearman correlation. To visualize the relation between GE and CN for the top-ten genes with the highest correlation in the Uppsala cohort for adenocarcinoma and squamous cell carcinoma, the box plots provided by cBioPortal were used.

### Gene ontology

Gene ontology group enrichment was performed using the topGO package [37] and Fisher’s exact test, and only results from the biological process ontology were considered. The resulting p-values were adjusted for multiple testing by the Benjamini−Hochberg procedure [38].

### Survival analysis and correlation with clinical parameters

A Cox proportional hazards model, according to Klein and Moeschberger [39], and the R package ‘survival’ [40], was used to determine the association with overall survival for significantly correlated CN-GE pairs based on CN and GE data. Overall survival (OS) was computed from the date of diagnosis to the date of death.

To validate significant survival associations in independent patient cohorts, the R package ‘meta’ [41] was applied to perform a meta-analysis of ten publicly available NSCLC data sets with Affymetrix HG U133A or Plus 2.0 array expression data and corresponding overall survival times (in total 1779 patients): GSE29013 [42], GSE30219 [43]; GSE31210 [44], GSE14814 [45], GSE19188 [46], GSE3141 [47]; Shedden et al., 2008 [48]; GSE4573 [49], GSE50081 [50] and GSE37745 [20]. All data sets were downloaded from the Gene Expression Omnibus (http://www.ncbi.nlm.nih.gov/geo/) except for Shedden et al. [48] which was downloaded from the website of the National Cancer Institute (https://array.nci.nih.gov/caarray/). The raw data was normalized using frozen robust multiarray analysis (fRMA) [51] apart from GSE4573 and GSE3141 for which only MAS5 normalized data was available. Normal (non-tumoral) samples and small cell carcinomas were removed. All data sets were checked for duplicates and a pair of patients was considered duplicates when the correlation of their expression values was ≥ 0.999. Between the dataset of Shedden et al. [48] and GSE14814, 43 duplicates were removed in the latter. In GSE37745 two different measurements for one patient were removed [52]. Meta-analysis was performed with random effects models based on the parameter estimates of log hazard ratios of the univariate Cox survival models and their standard errors. Inverse variance weighting was used to combine the single estimates to a pooled estimate. Significance of the overall effect was assessed by the p-value of the random effects model. Results were visualized with forest plots, in which parameter estimates of all single studies and the pooled estimates along with their confidence intervals are plotted on top of each other. All analyses were performed using R version 3.2.1 [53]. Multiple testing adjustments of significance levels were performed using the false discovery rate (FDR) [38]. Unadjusted p-values were considered as descriptive measures.

To further confirm the results of the survival analysis for the probesets that showed a significant prognostic impact in the meta-analysis using Affymetrix gene array data, Kaplan-Meier analysis was performed, using lung cancer RNA-seq data generated by the Cancer Genome Atlas (TCGA), via an easy-to-use interface provided by the Human Protein Atlas (http://www.proteinatlas.org, [54]). The unadjusted p-values based on the log-rank test, performed with an optimized cut-off as described [54], for lung adenocarcinoma and squamous cell cancer, as well as both combined, are reported.

## Results

### Visualization of genome-wide gene copy numbers and gene expression levels

**Fig 1:**
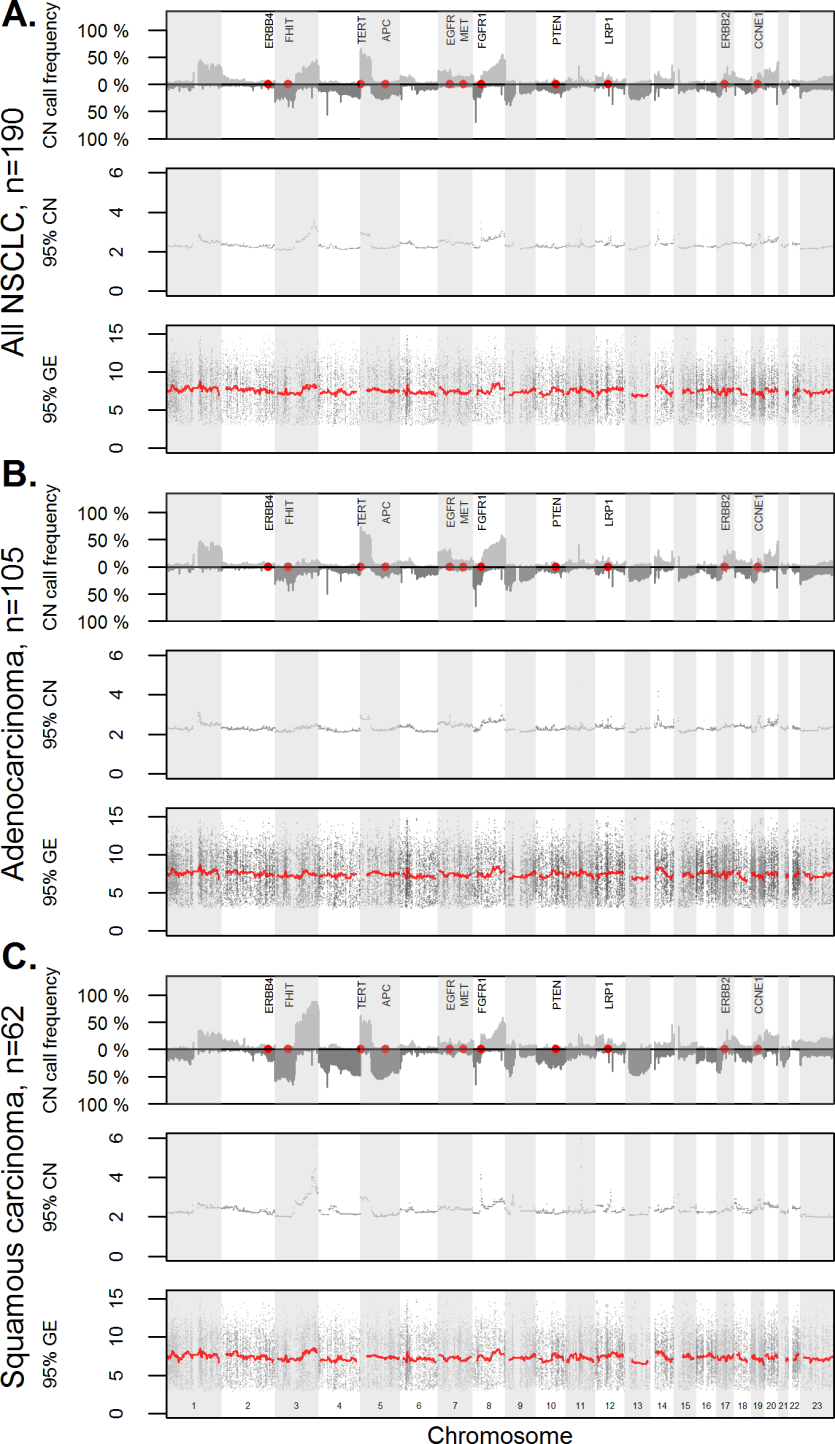
Gene copy number changes of non-small cell lung carcinomas. Copy number (CN) call frequencies are given for all NSCLC cases (A), adenocarcinomas (B) and squamous cell cancer (C). The frequency plots (upper graphs) give the proportion of cases with loss and gain for each chromosomal position. Positions of selected genes (ERBB4, FHIT, APC, EGFR, MET, PTEN, LRP1, ERBB2, CCNE1) were highlighted in red.

Tissue specimens of 190 NSCLC patients were analyzed for gene copy numbers (CN) and corresponding gene expression (GE) levels. First, the frequencies of genomic alterations, i.e. the percentage of patients with gain or loss, across the genome were illustrated for the complete NSCLC cohort, as well as for the histological subgroups of adenocarcinomas and squamous cell carcinomas (Fig 1A–1C, top panel). The observed pattern of gains and losses across the chromosomes corresponded well to the results of previous studies [4, 14–15, 18–19, 55–58], (S2 Table).

### Correlation of gene copy number and gene expression

**Fig 2:**
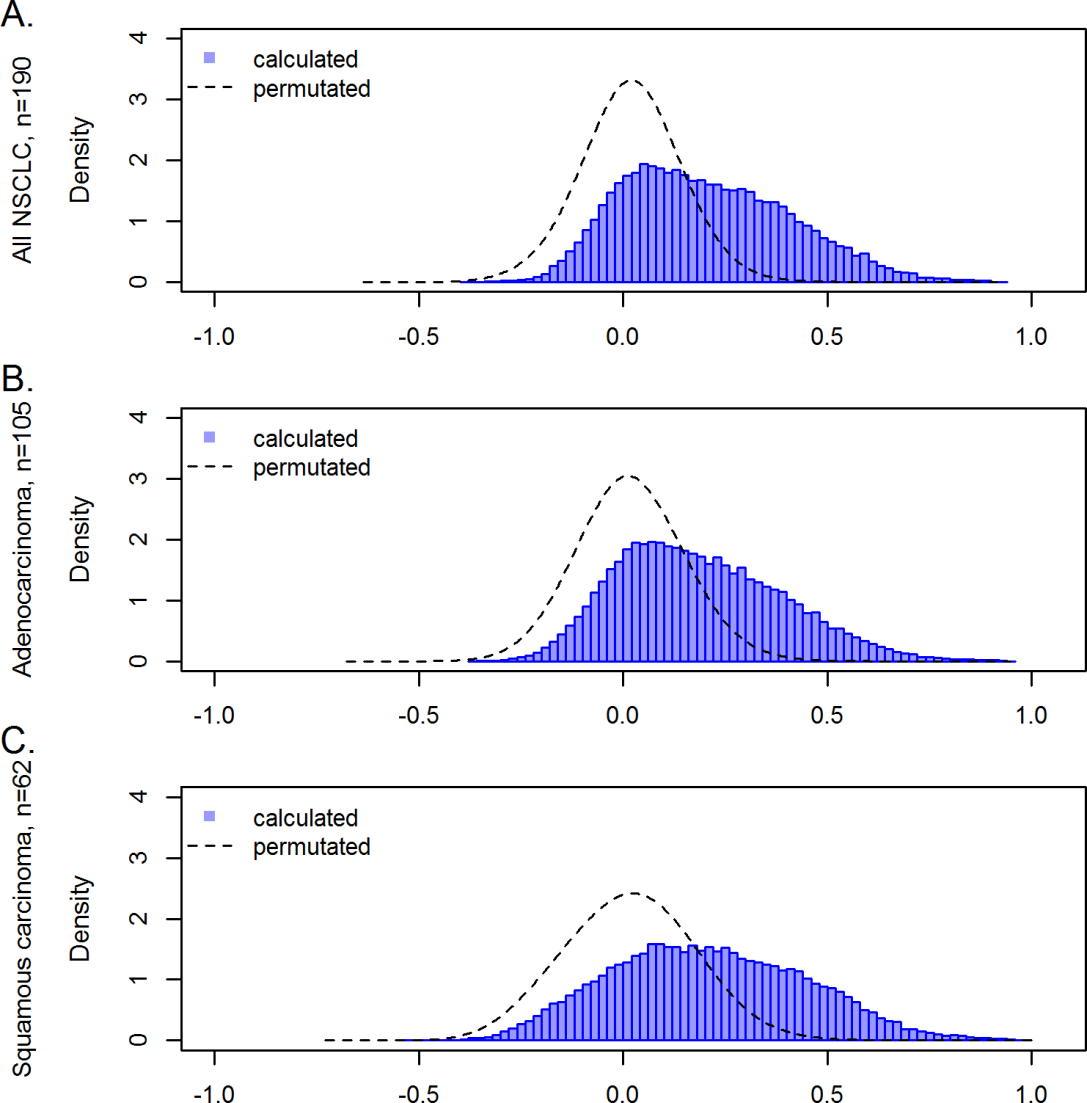
Correlation of gene copy number and corresponding gene expression values. Density analysis of the externally centered correlation coefficient (ECCC) log transformed (blue) mRNA expression values for all NSCLC cases (A), adenocarcinomas (B) and squamous cell cancer cases (C). The line forming a Gauss curve illustrates correlation values of randomly generated data. Randomization was achieved by permutation of gene copy number/mRNA pairs.

To systematically study the relationship between gene copy numbers and RNA levels, the gene expression (GE) value of each probe set and the corresponding gene copy number (CN) was identified. Matched CN and GE data were available for 39,788 probe sets, corresponding to 18,756 genes. Correlation between these pairs of CN and GE was analyzed by the externally centered correlation coefficient (ECCC). The ECCC frequency distribution of all CN-GE pairs showed a shift towards positive values compared to permutated (random) data (Fig 2A–2C). This shift to positive correlation coefficients is in line with the intuitive notion that copy number gain may lead to higher RNA levels or copy number loss to lower expression. In contrast, inverse correlations between CN and GE are not observed to a higher degree than randomly expected, as illustrated by the dashed line of permutated data (Fig 2). A statistically significant correlation (FDR adj. p<0.05) between CN and GE was observed for 19,058 probe sets (10,296 genes) (Table A in S3 Table). Note that already very small ECCC values may result in p-values smaller than 0.05. Since the biological relevance of very small correlation coefficients is questionable, we focused on CN-GE pairs with a high ECCC. Considering copy number gain and increased gene expression, correlation coefficients (ECCC) were higher than 0.5 for 3,482 probe sets (1,351 genes). Correlations with ECCC higher than 0.7 were observed for 440 probe sets (301 genes). The highest ECCC was 0.927 (Table A in S3 Table). The 25 probe sets with the highest ECCCs are listed in Table 1.

**Table 1:** Top 25 probe sets with highest ECCC of gene copy number levels and mRNA expression for tumors from 190 NSCLC patients.

gene symbol	gene	chromosome	probe set	ECCC*	FDR^#^	frequency of gain (in %)
CDCA4	cell division cycle associated 4	14q32.33	218399_s_at	0.93	0.0166	15.26
EAPP	E2F-associated phosphoprotein	14q13.1	202623_at	0.93	< 0.0001	19.47
KIAA0391	KIAA0391	14q13.2	202713_s_at	0.93	< 0.0001	19.47
BTBD6	BTB (POZ) domain containing 6	14q32.33	225389_at	0.92	< 0.0001	15.26
SRP54	signal recognition particle 54kDa	14q13.2	203605_at	0.92	< 0.0001	18.95
PACS2	phosphofurin acidic cluster sorting protein 2	14q32.33	212778_at	0.92	< 0.0001	15.26
PACS2	phosphofurin acidic cluster sorting protein 2	14q32.33	34406_at	0.92	< 0.0001	15.26
MTA1	metastasis associated 1	14q32.33	211783_s_at	0.92	< 0.0001	15.26
NXT1	nuclear transport factor 2-like export factor 1	20p11.21	218708_at	0.90	< 0.0001	26.84
BAZ1A	bromodomain adjacent to zinc finger domain, 1A	14q13.2	217986_s_at	0.90	< 0.0001	19.47
KRAS	Kirsten rat sarcoma viral oncogene homolog	12p12.1	204009_s_at	0.90	< 0.0001	13.16
KAT6A	K(lysine) acetyltransferase 6A	8p11.21	226547_at	0.89	< 0.0001	13.68
GEMIN2	gem (nuclear organelle) associated protein 2	14q21.1	205063_at	0.89	< 0.0001	16.84
GEMIN2	gem (nuclear organelle) associated protein 2	14q21.1	211115_x_at	0.89	< 0.0001	16.84
BAZ1A	bromodomain adjacent to zinc finger domain, 1A	14q13.2	217985_s_at	0.89	< 0.0001	19.47
NAPB	N-ethylmaleimide-sensitive factor attachment protein, beta	20p11.21	225111_s_at	0.89	< 0.0001	26.32
SNX6	sorting nexin 6	14q13.1	217789_at	0.89	< 0.0001	19.47
CST3	cystatin C	20p11.21	237623_at	0.88	0.0014	26.32
CNOT2	CCR4-NOT transcription complex, subunit 2	12q15	217798_at	0.88	< 0.0001	13.16
GZF1	GDNF-inducible zinc finger protein 1	20p11.21	225884_s_at	0.88	< 0.0001	26.84
GEMIN2	gem (nuclear organelle) associated protein 2	14q21.1	211114_x_at	0.88	< 0.0001	16.84
GEMIN2	gem (nuclear organelle) associated protein 2	14q21.1	210779_x_at	0.88	< 0.0001	16.84
EGFR	epidermal growth factor receptor	7p11.2	201984_s_at	0.88	< 0.0001	28.42
SLC35E3	solute carrier family 35, member E3	12q15	218988_at	0.88	< 0.0001	12.11
NAPB	N-ethylmaleimide-sensitive factor attachment protein, beta	20p11.21	1570441_at	0.88	0.0004	26.32

* ECCC = externally centered correlation coefficient

^#^ FDR = false discovery rate

In a next step, the correlation analysis was performed separately for the adenocarcinoma and squamous cell carcinoma histological subtypes (Tables B and C in S3 Table). For adenocarcinomas, 13,282 probe sets (7,897 genes) showed a significant correlation between CN and GE; 383 probe sets (269 genes) with ECCC>0.7 (Table B in S3 Table). For squamous cell carcinomas, the corresponding numbers were 9,560 probe sets (6,014 genes) and 626 probe sets (465 genes), respectively (Table C in S3 Table). For most genes, a strong correlation was observed in one subtype only (adenocarcinoma: 219; squamous cell cancer: 415), while only 50 genes showed a strong correlation in both histologic entities.

Only 178 probe sets (113 genes) showed a significant correlation (FDR adj. p<0.05) between copy number loss and decreased gene expression, whereby copy number loss was defined by a median copy number below 1.9 and a 25% quantile below 1.5. These correlations were relatively weak (only seven probe of them reached an ECCC higher than 0.4) and were not further analyzed in this study.

### High copy number and high gene expression is associated with higher correlation coefficients

**Fig 3:**
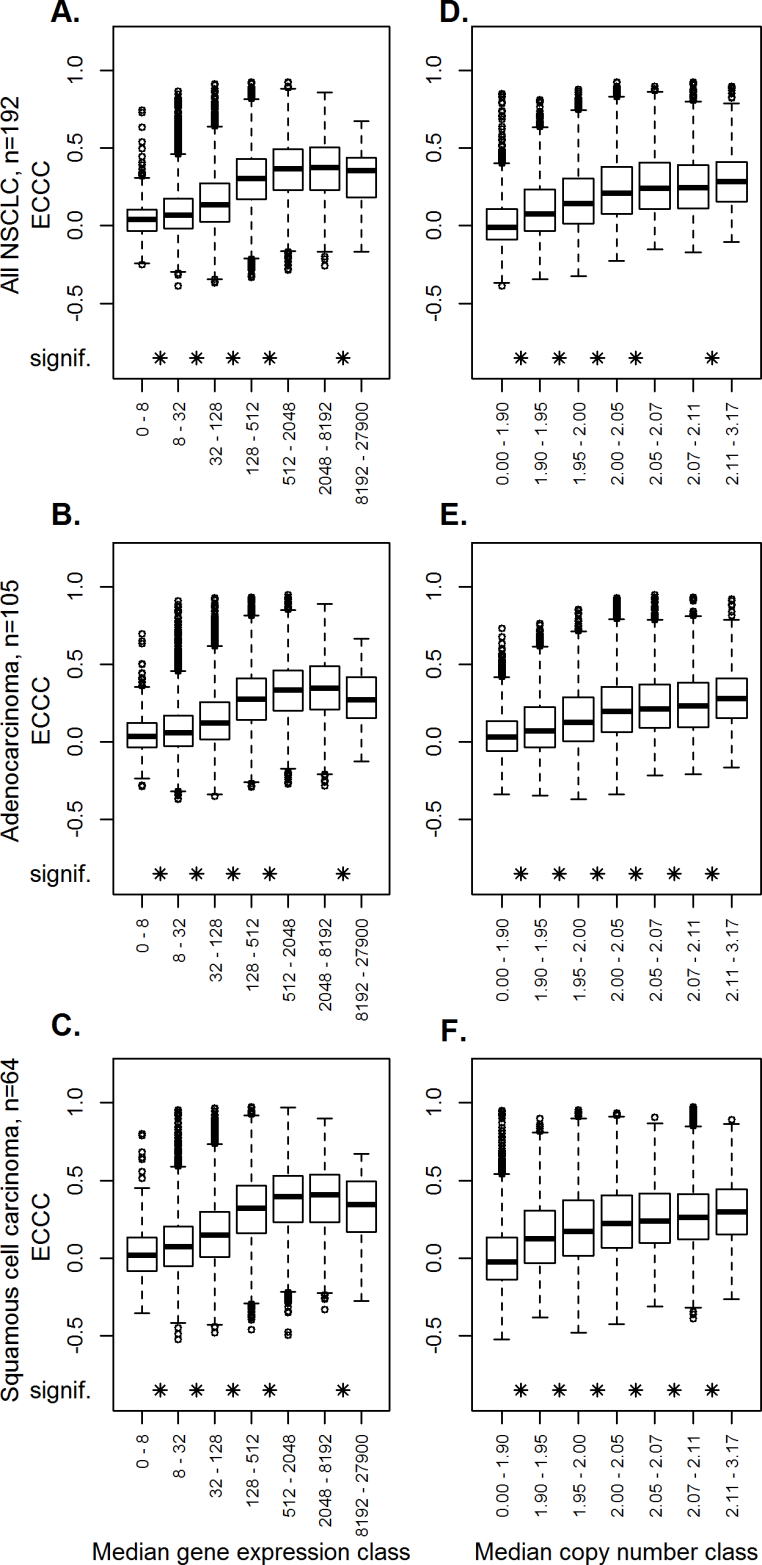
Dependency of the externally centered correlation coefficient of gene expression levels and gene copy numbers. Influence of gene expression levels. Probe sets were subdivided into seven classes, based on quantiles, according to their median gene expression levels. The boxplot represents the externally centered correlation coefficients for all probe sets within each class (A-C). Influence of copy numbers. Probe sets were subdivided into seven classes, based on quantiles, according to their median gene copy number. The boxplot represents the externally centered correlation coefficients for all probe sets within one class (D-F). The analyses were performed for all NSCLC (A, D), adenocarcinomas (B, E) and squamous cell cancer (C, F). The asterisks indicate significantly different ECCC values compared to the next lower group.

We next focused on factors that might have an impact on the correlation between copy number and gene expression. First, we analyzed a possible association of gene expression levels on ECCC. Comparing percentiles of probe sets with increasing gene expression, higher median gene expression levels were associated with higher ECCCs (Fig 3A–3C), and significant differences between individual groups and the next were indicated after FDR adjustment (Wilcoxon test, FDR adj. p<0.05). To then determine the influence of median gene copy number on the ECCC, percentiles of probe sets with increasing copy numbers were formed, illustrating a significant association of correlation coefficients (ECCC) and median copy number (Wilcoxon test, FDR adj. p<0.05 for each group tested the following) (Fig 3D–3F).

### Relatively small numbers of tumors with high expression and high copy numbers explain most of the high correlation coefficients

In the further analyses we focused on the 440 probe sets (301 genes) with high correlation coefficients (ECCC>0.7; FDR adj. p<0.05) identified in the analysis of the complete cohort (i.e. including all histological tumor types), hereafter referred to as “*highly correlating probe sets”* and, correspondingly, *“highly correlating genes”*. To visualize the relationship between gene copy number and gene expression of the 440 highly correlating probe sets, scatter plots with gene expression on the y-axis and gene copy number on the x-axis were generated (S1 Fig). A typical feature in many of these scatter plots is that only a relatively small fraction of tumors shows high copy numbers and high expression values, which explains the high correlation coefficients.

**Fig 4:**
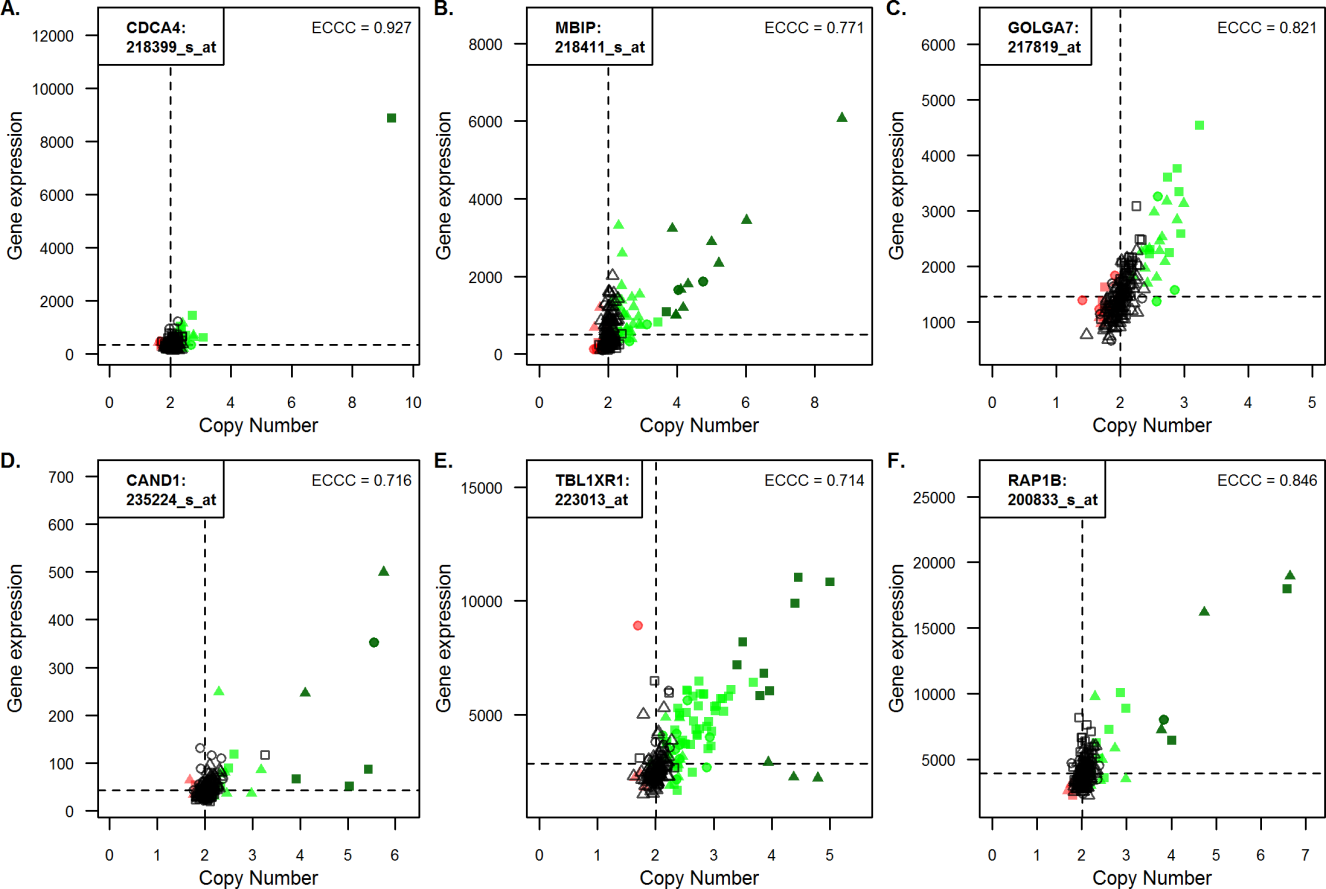
Relationship between gene copy number and corresponding gene expression values for selected probe sets. Gene expression values in a linear scale were plotted against gene copy number values (also linear) for all 190 NSCLC cases for selected genes. Triangle and square symbols represent adenocarcinomas and squamous cell cancer, respectively, while the circles stand for undifferentiated large cell carcinomas. Dark green represents a high gain, green represents gain and red copy number loss. The correlation is given as externally centered correlation coefficient (ECCC). Abbreviated gene names and the accession numbers of the corresponding probe sets are given in the left corner of each panel.

For 394 of the 440 probe sets (90%), at least one tumor exhibited high copy number gain. The following constellations were frequently observed (Fig 4A–4C): for 79 highly correlating probe sets (56 genes), only one tumor displayed high copy number gain as illustrated for the example of CDCA4 (Fig 4A); for 29 highly correlating probe sets (14 genes), more than ten tumors showed high copy number gain, exemplified by in Fig 4B (ECCC = 0.771); and for 46 highly correlating probe sets (40 genes), no tumor demonstrated high copy number gain (Fig 4C).

As shown in the section about correlation of gene copy number and gene expression, correlation can be tumor subtype specific. CAND1 represents an example, where high CN gain is associated with increased gene expression in adenocarcinomas but not in squamous carcinomas (Fig 4D). The opposite constellation was observed for example for TBL1XR1 (Fig 4E). RAP1B represents an example where high CN gain of both adeno- and squamous carcinomas is associated with high gene expression (Fig 4F).

**Fig 5:**
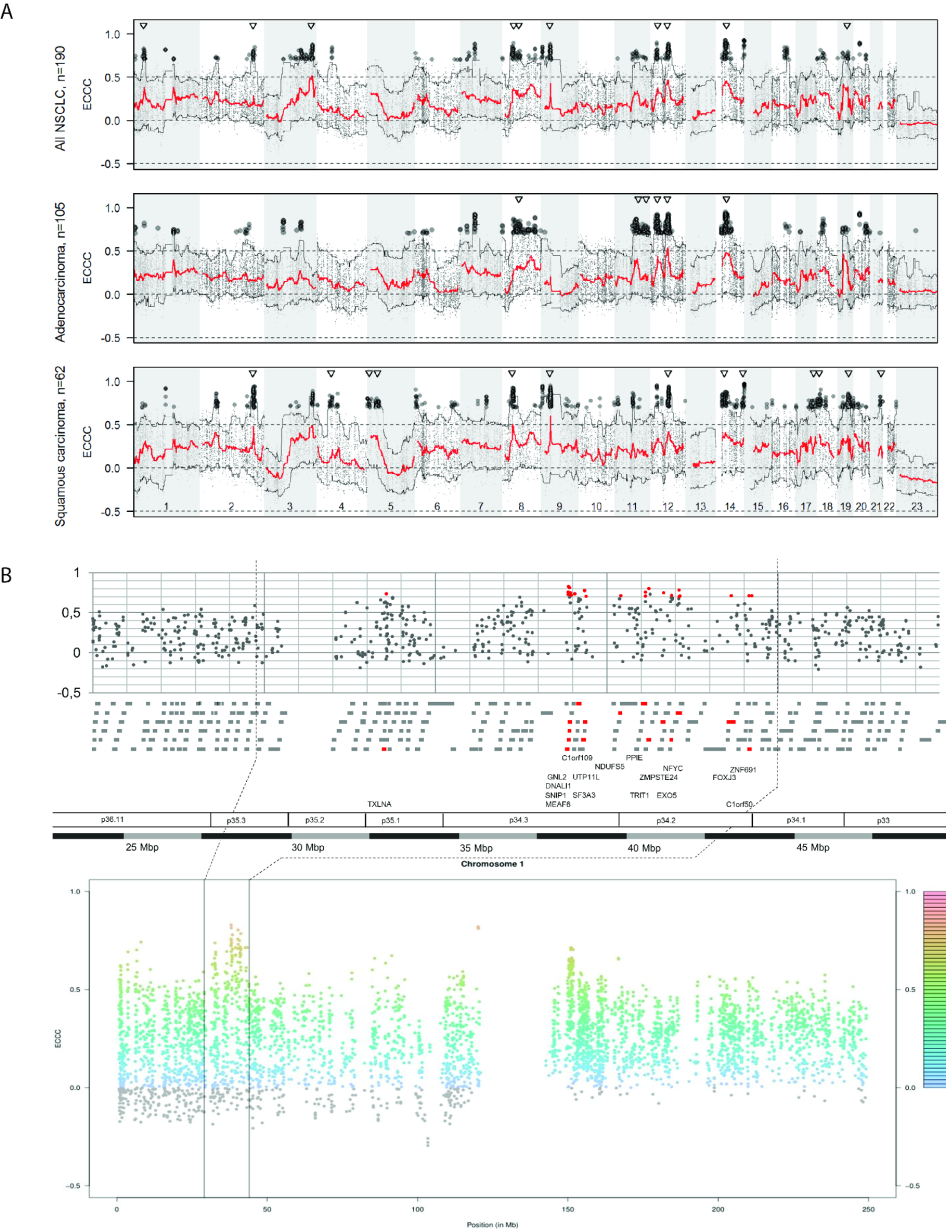
Correlation between gene copy number and corresponding gene expression values over all chromosomes. (A) The modified Manhattan plot gives the median (red line) of the externally centered correlation coefficient (ECCC) and the 5% and 95% quantile (black lines) along all chromosomal positions. The black dots indicate the probe sets with highest ECCC (ECCC>0.7, FDR adj. p<0.05). The triangles show the position of hotspot regions. High correlation between copy number variation and gene expression is not randomly distributed over the genome but mostly occurs within specific chromosomal regions (hotspots). (B) Example of the hotspot region on chromosome 1p35-1p34 comprising 185 genes (gray bars) with 17 highly correlating genes (red bars). Each dot represents the ECCC of a probe set based on the analysis of all 190 NSCLC cases.

Visual inspection of modified Manhattan plots indicated that the highly correlating genes appeared to cluster in distinct chromosomal regions (Fig 5A). To objectify these regions, we applied moving windows of different genomic sizes (5, 10, 15, 20 and 50 Mbp) and scanned the genome for regions with at least ten highly correlating genes per window (“hotspots”). With a width of 15 Mbp we identified ten regions of increased density of highly correlating genes, including between 14 and 48 highly correlating probe sets (11–29 genes, in total 169 genes) (S4 Table). To illustrate a representative pattern of highly correlating genes, a region on chromosome 1p34 was selected (Fig 5B). This chromosomal region comprised 18 genes (23 probe sets) with strong correlations highlighted by red color. The dotted vertical lines indicate the position of the 15 Mbp window in the upper and lower panel (Fig 5B). The indicated region on chromosome 1p34 illustrates that the density of highly correlating genes is higher within p34.2 and p34.3 compared to the up- and downstream neighborhood. Chromosomal positions of these regions on chromosomes 3, 8, 9, 12, 14 and 19 coincide with high values of the 95% quantiles of the segmented gene copy numbers (S2 Fig). This is in agreement with the aforementioned observation that high correlation coefficients (ECCC) are typically obtained for genes with high copy numbers in some patients and that high copy number is associated with higher ECCC (Fig 3).

### Enrichment of biological motifs among highly correlating genes

Next, we analyzed if certain biological motifs were overrepresented among highly correlating genes. Twenty-four significantly overrepresented gene ontology (GO) terms (FDR adj. p<0.05) were identified (Table A in S5 Table). Among these, GO groups associated with posttranslational protein modifications were particularly frequent. Altogether, 27 highly correlating genes were involved in ubiquitination (GO:0016567, GO:0032436, GO:0010265), neddylation (GO:0045116, GO:0010265) and protein destabilization or catabolic processing (GO:0031648; GO:1903363). A well-known highly correlating gene in these motifs is MDM2, an oncogene and an E3 ubiquitin-protein ligase, which targets the tumor suppressor p53, and was represented by five probe sets (ECCC 0.78–0.85). Also five additional E3 ubiquitin-protein ligases (RNF38, SIAH1, SIAH2, HECTD1, G2E3) were identified by the GO enrichment analysis.

For the highly correlating genes identified in the adenocarcinoma and squamous cell carcinoma subgroups, respectively, we identified *inter alia* significantly overrepresented GOs associated with DNA repair (GO:0045739, GO:0010569) and embryonal development (GO:0001824) in the adenocarcinomas, and GOs related to apoptosis (GO:0042771) in squamous cell cancer (FDR adj. p<0.05) (Tables B and C in S5 Table).

### Gene copy number/gene expression correlations are higher for ‘cancer genes’

Numerous oncogenes and tumor suppressor genes play a role in NSCLC development and progression. For some of them, copy number aberrations are considered to be involved in their activation or inactivation. To evaluate whether genes with a known link to cancer show high correlations between copy number and gene expression, the ECCC of 533 ‘cancer genes’ (1728 probesets; Table A in S6 Table) defined by the Cancer Gene Census (http://cancer.sanger.ac.uk/cancergenome/.projects/census/, [34]) were compared to those of all other genes. The ‘cancer genes’ were found to exhibit higher ECCC (p<0.001; Wilcoxon test; Figs A-C in S3 Fig). The association between the 533 ‘cancer genes’ and high ECCC may be due to the above described association of ECCC with higher expression as well as higher copy number. Therefore, a multivariate model was applied where the difference between ‘cancer genes’ versus all other genes was stratified for copy number and gene expression. For adjustment, the 95% percentile of copy number and gene expression was used, since the significance of correlations (ECCC) often depends on a relatively small fraction of patients with high copy number and high expression values. When adjusted for both, copy number and gene expression, the difference in ECCC between ‘cancer genes’ and all other genes was no longer significant (p = 0.788).

Next we focused on 43 genes (156 probe sets) that previously have been described as overexpressed or amplified in NSCLC (Table B in S6 Table). The ‘lung cancer genes’ were found to exhibit higher ECCC compared to all other genes (p<0.034; Wilcoxon test; Figs D-F in S3 Fig). For 31 of the ‘lung cancer genes’ (86 probe sets), at least one probe set displayed a significant correlation between copy number and gene expression, and for 3 genes (11 probe sets) at least one probe set displayed a median ECCC>0.7. The latter are the well-established NSCLC driver genes, *KRAS* (ECCC 0.71–0.90; 3 probe sets) and EGFR (ECCC 0.71–0.88; 3 probe sets), as well as the p53-targeting E3 ubiquitin ligase MDM2 (ECCC: 0.78–0.85; 5 probe sets). However, in a multivariate model the difference in ECCC of the 43 ‘lung cancer genes’ and all other genes was no longer significant after stratification for copy number and gene expression (p = 0.24). For the histologic subtypes, the corresponding adjusted analyses led to a significant difference for squamous cell carcinoma (p = 0.016) but not for adenocarcinoma (p = 0.172).

### Highly correlating genes are enriched among survival-associated genes

**Fig 6:**
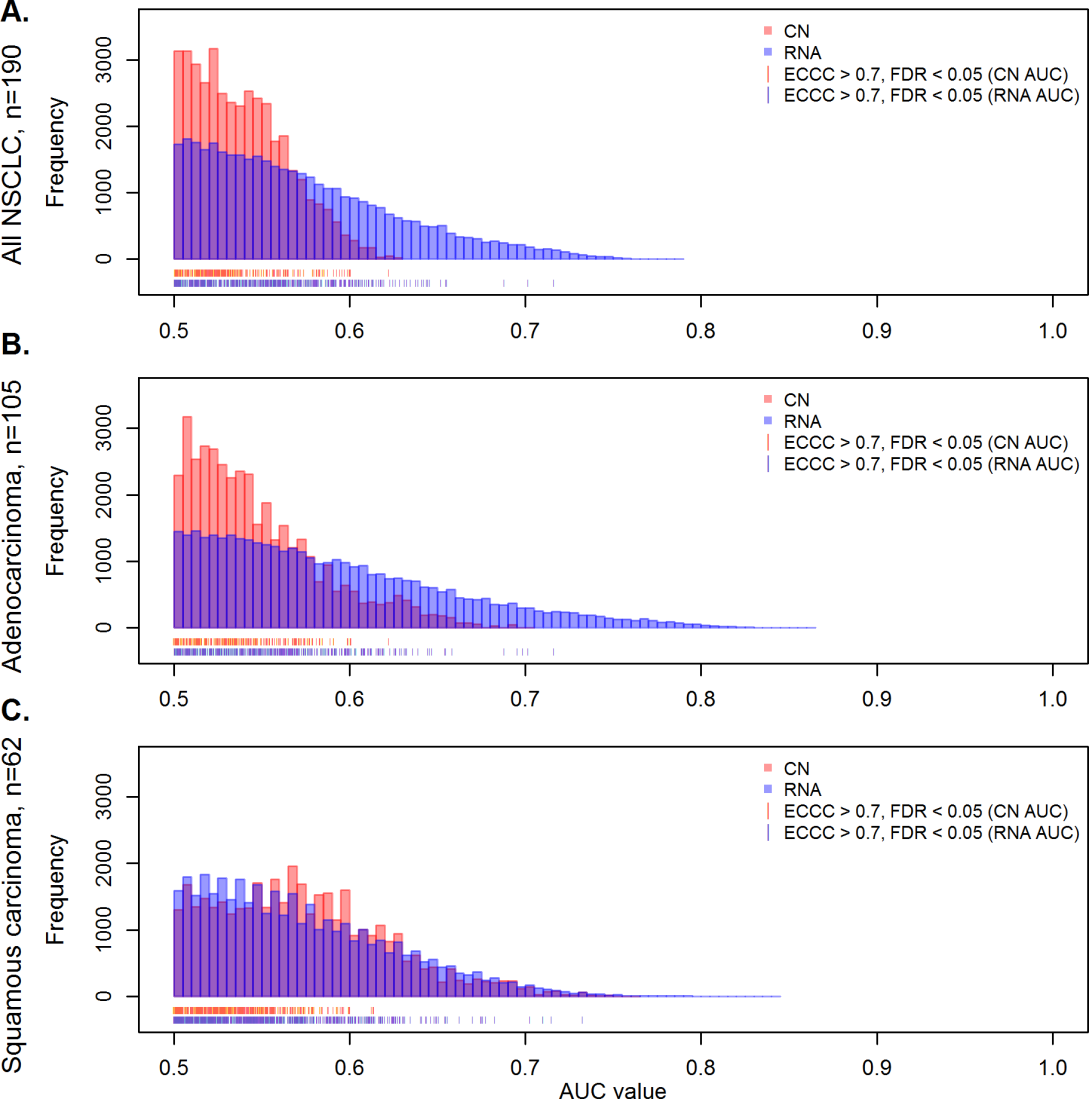
Impact of gene copy number and gene expression on survival. The plot shows the number of probe sets in relation to the AUC as a measure of survival association for gene copy number (red) and gene expression values (blue) in the complete set of NSCLC (A), adenocarcinomas (B) and squamous cell cancer (C). Considering all 39,788 matched copy number/RNA pairs it is evident that RNA levels exert a higher prognostic impact than gene copy number in the total group and in adenocarcinomas, while a similar influence of RNA levels and gene copy number is seen in squamous carcinomas. The red and blue strokes at the x-axis represent genes with highest externally centered correlation coefficients (ECCC>0.7, FDR adj. p<0.05).

To obtain a genome-wide overview of survival associations, we first determined for each gene how gene expression, and gene copy number levels were able to separate between patients surviving shorter and longer than two years after diagnosis by calculating the area under the receiver operating characteristic (ROC) curve (AUC). Considering all 39,788 matched copy number-probe set pairs, gene expression discriminated better between long- and short-term survivors. This is illustrated by the AUC-value distribution that shifted towards higher values for gene expression (RNA) as compared to gene copy number when all NSCLC were analyzed (Fig 6A). A similar constellation as for the total group was also obtained for the adenocarcinomas (Fig 6B). However, for squamous cell carcinomas the two frequency distributions were overlapping, illustrating that in this histological subtype gene copy number and gene expression separate similarly well between patients surviving shorter and longer than two years (Fig 6C).

Next, we focused on the highly correlating probe sets (ECCC>0.7, FDR adj. p<0.05), denoted by red and blue lines below the AUC-values histogram (Fig 6A–6C). None of the 440 highly correlating probe sets showed a significant association with survival after false discovery rate adjustment in the uni- and multivariate analysis in the complete cohort as well as in the adeno- and squamous carcinomas (S7 Table). However, the case number of 190 in the present cohort may have been too low to allow identification of prognostic significance under conditions of false discovery adjustment. Therefore, the 440 highly correlating probe sets were analyzed in a meta-analysis of ten publicly available gene expression cohorts with totally 1,779 patients, including also the Uppsala cohort. In total 70 of the 440 highly correlating probe sets (16%) revealed a significant association with survival (FDR adj. p<0.05) in the meta-analysis of all NSCLC (Table A in S8 Table). This is a significantly higher number than randomly expected considering all prognostic probe sets that are represented on the microarray (2,843 of 39,348 probe sets, p<0.002, Fisher-test). Thus, survival associated probe sets are significantly overrepresented among the 440 highly correlating probe sets (results of the histological subgroups in Tables B and C in S8 Table).

**Fig 7:**
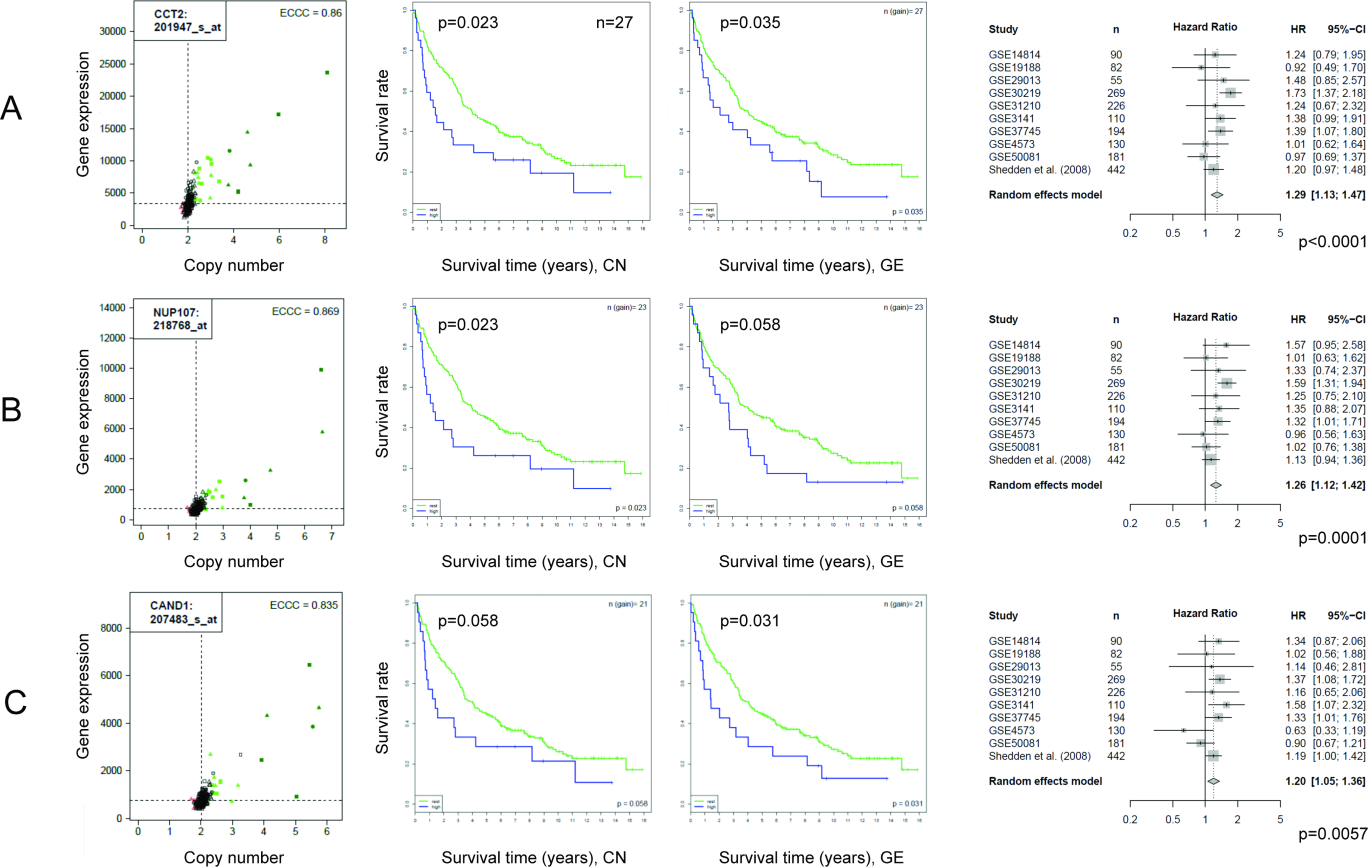
Highly correlating genes and association with survival. The meta-analysis of 10 NSCLC cohorts revealed that 70 of 440 highly correlating genes were associated with survival. For illustration three of the 70 genes are shown: CCT2, NUP107 and CAND1. The correlation between copy number and gene expression is shown by scatter plots (left). Survival time is visualized by Kaplan Meier plots (two panels in the middle). Patients were dichotomized at the 75% percentile for copy number (middle, left) and gene expression (middle, right). The forest plots illustrate the results of the meta-analysis including nine independent data sets and the Uppsala cohort (right).

Three examples of genes that showed strong correlations between copy number and gene expression with ECCC>0.7 and were significantly associated with survival based on gene expression level information in the meta-analysis of 1,779 non-small cell lung cancer patients are shown in Fig 7: Chaperonin Containing TCP1, Subunit 2 Beta (CCT2); Nucleoporin 107kDa (NUP107), a component of the nuclear pore complex; and Cullin-Associated And Neddylation-Dissociated 1 (CAND1), which plays a role in protein ubiquitination and neddylation. For all three genes also the Kaplan Meier plots for the Uppsala cohort are shown, differentiating between patients with copy number gain versus non-gain as well as low versus high gene expression. In contrast to the meta-analysis, significance was not reached for all analyses when the Uppsala cohort was analyzed alone, probably due to the relatively low case number of 190 patients but the trend was that high expression is associated with worse prognosis, similarly as for the meta-analysis (Fig 7).

### External validation using TCGA data

As a final point, matched CN and GE data generated by the Cancer Genome Atlas (TCGA) were used to study whether the results could be confirmed in an independent cohort. Firstly, TCGA data from 520 adenocarcinomas and 504 squamous cell cancers were used to study the correlation between CN and GE (S9 Table). The analysis of the highly correlating genes of the Uppsala cohort resulted in Spearman correlation coefficients of >0.7 for 7%, >0.6 for 29% and >0.5% for 63%, when adenocarcinomas and squamous carcinomas were analyzed together (Table A in S9 Table), and similar concordance were obtained when the analysis was performed for the two histological entities separately (Tables B and C in S9 Table, S4 Fig).

Secondly, the association with survival in the TCGA RNA-seq data set was analyzed for the 70 highly correlating probe sets that were associated with survival in the meta-analysis. In the combined analysis of both adenocarcinomas and squamous cell carcinomas, 23%, 49% and 69% of the probe sets showed unadjusted p-values < 0.01, 0.05 and 0.10 (log-rank test), respectively (Table A in S10 Table). The corresponding FDR adjusted p-values are also given in Table A in S10 Table. The percentage of genes with significant p-values < 0.01, 0.05 and 0.10 were 57%, 86%, and 90%, respectively, when the adenocarcinomas were analyzed separately (Table B in S10 Table).

In conclusion, the correlation between CN and GE, as well as the association with survival observed for a high fraction of the highly correlating genes in the meta-analysis, could be confirmed in an independent cohort from the TCGA.

## Discussion

This study provides a genome-wide analysis of gene copy number changes and corresponding gene expression levels in 190 NSCLC patients. Genome-wide analysis of copy number-gene expression pairs revealed the following features: (1) While more than half of all analyzed gene copy number-gene expression pairs showed a statistically significant correlations in the analyzed cohort (10,296 of 18,756 genes), strong correlations with ECCC>0.7 were obtained only in a small subset of 301 genes (1.6%, represented by 440 probe sets), suggesting that only for a minority of genes variability of gene expression is strongly associated with copy number variation. One explanation for the relatively low number of strong correlations is that many genes show no or only very weak increases in copy number in the analyzed tumors. Variability in copy number of a given gene is a precondition for a correlation with gene expression. Typical examples, where high copy number gain correlates with high gene expression are MBIP, CAND1, TBL1XR1 and RABP1B. (2) Higher correlation coefficients were associated with higher copy number and expression levels. Recently, it has been reported that abundantly expressed genes are more gene dosage sensitive than genes with low overall expression levels [59], in agreement with the results of our study. (3) Strong correlations were frequently based on only a few tumors with high copy number gain and correspondingly increased gene expression. (4) 24 significantly overrepresented gene ontology (GO) terms (FDR adj. p<0.05) were identified among the highly correlating genes. Among them GO groups associated with posttranslational protein modifications were particularly frequent, including ubiquitination and neddylation. While ubiquitination is involved in most cellular processes, neddylation is considered to be more specific with conjugation of the ubiquitin-like protein NEDD8 to cullin family members and some further target proteins [60]. Recent evidence suggests that protein ubiquitination and neddylation are involved in DNA damage response [60] and thus deregulation may occur in response to malignant transformation or may be actively involved in carcinogenesis. Notably, genes involved in the neddylation pathway are upregulated in lung cancer and protein expression in adenocarcinomas has been reported to be associated with worse prognosis [61]. (5) Highly correlating genes were overrepresented among survival associated genes. To study the association of gene expression and survival with sufficient statistical power, we performed a meta-analysis with totally 1,779 patients, including the here analyzed Uppsala cohort. This study demonstrated, based on gene expression data, that 70 of the identified highly correlating genes demonstrated an impact on prognosis.

Only few other studies systematically integrated gene copy number changes and gene expression in a genome-wide analysis of clinical lung cancer samples. One of the first studies evaluated nine squamous cell cancer samples with complementary data from custom made BAC-CGH arrays and Agilent gene expression microarrays [14]. With overlay-tools the authors identified that mRNA levels of more than 2,000 genes were explained by gene copy number changes. The study of Lazar and colleagues integrated gene copy number alterations, gene expression and microRNA profiles to explore cancer markers to distinguish between adenocarcinoma and squamous cell cancer for clinical purpose [15]. MRPS22, NDRG1 and RNF7 were found to be consistently over-expressed in amplified genomic regions in agreement with a strong correlation observed in our dataset (ECCC = 0.81, 0.42 and 0.79 for MRPS22, NDRG1 and RNF7, respectively). In the study of Gallegos Ruiz, 32 NSCLC cancer samples were analyzed by a gene expression microarrays and in house CGH arrays, detecting 359 transcripts, corresponding to 248 genes that were significantly affected by gene copy number changes [17]. Of these genes, 25 (KPNA4, DHX36, ATP1B3, PHC3, PDCD10, SIAH2, GMPS SLC25A36, RNF7, TBL1XR1, ASTE1, TFDP2, ABCC5, ATR, COPB2, PIK3R4, MRPS22, B3GNT5, ZNF639, MCCC1, DCUN1D1, ATP11B, DNAJC19, FXR1, MFN19) were also in our list of highly correlating genes.

Recently, it was pointed out that integrative cancer studies that focus on genes whose expression levels correlate with corresponding copy numbers might create a bias towards genes whose expression levels are not strongly controlled by non-genetic regulatory processes [59]. We agree that strongly regulated genes are of high interest when one aims to understand global relationships between copy number and gene expression. However, one goal of the present study was to identify genes for which expression strongly correlates with copy number and that also are associated with survival. The strong DNA-RNA correlation clinical relevance, combined with might indicate a functional relevance or better define stable molecular subgroups of patients. To identify such genes, we applied the criteria of high correlation between copy number and gene expression (ECCC>0.7) in the Uppsala cohort, and association with survival in a meta-analysis, including data from 1,779 non-small cell lung cancer patients with an FDR adjusted p-value smaller than 0.05. As much as 70 genes fulfilled these criteria, among them the chaperone CCT2, the core complex protein NUP107 and the ubiquitination and neddylation associated protein CAND1. CCT2 was recently identified as significantly depleted in an RNAi-based growth and viability screen, as well as copy number amplified and overexpressed in the SUM-52 breast cancer cell line [62]. Validation of the role of CCT2 in cell growth was provided by knocking down CCT2 using five shRNA constructs targeting CCT2. Moreover, a genomic region containing CCT2 was reported to be amplified in breast cancer, and further shown to be amplified and/or overexpressed in approximately 13% of human breast cancers analyzed as part of the Tumor Genome Atlas (TCGA) project. In the TCGA breast cancer dataset, CCT2 amplification and/or overexpression was associated with worse outcome [62], in agreement with studies in gall bladder carcinoma [63] and with our current findings in NSCLC.

To validate the main findings of the present study, an independent cohort (TCGA) was analyzed. The correlation of CN with GE, as well as the association of GE with survival for many of the highly correlating genes, could be confirmed for approximately half of the highly correlating genes. This finding may be relevant, because RNA levels for the highly correlating genes can be estimated based on copy number analysis. Copy number analysis can be performed with DNA which in contrast to RNA can easily be obtained from FFPE tissue. Despite of the significant association of the identified genes with survival it should be noted that their practical usefulness in clinical routine should be discussed with caution. Limitations of molecular prognostic biomarkers were recently demonstrated in a study testing the value of a panel of 5 immunohistochemical biomarkers [64] to predict NSCLC patient survival. Although the highly optimized panel showed a strong association with prognosis alone, it did not add significant prognostic information to the combination of traditional clinical factors (age, stage and performance status). Thus, it remains to be analyzed whether the here identified highly correlating prognostic genes add independent information to the clinical factors.

In conclusion, this study provides a genome-wide profile of gene copy number dependent gene expression in NSCLC, a description of highly correlating genes with biological motifs as well as chromosomal locations and finally a thorough analysis of their prognostic impact.

## Supporting information

S1 Fig Relationship between gene copy number and corresponding gene expression values for 440 highly correlating probe sets. Correlation analysis using the externally centered correlation coefficient (ECCC) revealed 440 probe sets with significant correlations (FDR adj. p < 0.05) and high correlation coefficients (ECCC > 0.7). Gene expression values in a linear scale were plotted against gene copy number values (also linear scale) for all 190 NSCLC cases. Triangular and square symbols represent adenocarcinomas and squamous cell cancer, respectively, while the circular symbols are undifferentiated large cell carcinomas. Dark green represents a high gain, green represents gain and red copy number loss. The correlation is given as externally centered correlation coefficient (ECCC). Abbreviated gene names and corresponding accession numbers of the probe sets are given in the left corner of each plot.
(PDF)

S2 Fig Chromosomal positions of regions with high CN-GE correlations (“hotspots”) and copy number values. The total cohort with 190 patients is shown. The panels give copy number (CN) call frequencies (from Fig 1), the externally centered correlation coefficients (ECCC) (triangles: “hotspot regions” of high correlation), the 95% quantile of the segmented gene copy number values (95% CN), and the 95% quantile (grey dots) and the moving average (red line) of the logarithmic gene expression values. The eight ECCC hotspot regions on chromosomes 3, 8, 9, 12, 14 and 19 coincide with higher values of the 95% CN.
(PPTX)

S3 Fig Analysis of cancer genes. Probe sets for cancer genes (n = 522) were selected based on Cancer Gene Census (Futreal et al. 2004) and the externally centered correlation coefficients (ECCC) were compared to the ECCC of all other probe sets (A-C). In addition, 152 probe sets for 46 genes were listed that previously have been reported to show genomic gain in NSCLC (D-F).
(JPG)

S4 Fig Visualisation of top 10 genes with the highest correlation in an independent data set. The box plots were based on gene copy numbers (estimated by GISTIC 2.0) and gene expression data from 520 adenocarcinomas and 504 squamous cancer cases provided by the TCGA were downloaded from cBioPortal (August 2017; http://www.cbioportal.org, Gao et al. Sci. Signal. 2013 & Cerami et al. Cancer Discov. 2012).
(PDF)

S1 Table Patient characteristics. Patient characteristics of 190 patients with available fresh frozen tissue. Tumor tissue was used for concurrent analysis of genome wide gene copy number and mRNA expression levels.
(DOCX)

S2 Table Genomic aberrations in the Uppsala study cohort compared to previous results. The table lists gains or losses observed in the present study (Uppsala) and indicates whether similar aberrations in the same chromosomal region have also been reported in previous studies.
(DOCX)

S3 Table Correlation of gene copy numbers and gene expression. (A) Correlation of gene copy numbers and gene expression values for 190 NSCLC cases. (B) Correlation of gene copy numbers and gene expression values for 105 adenocarcinoma cases. (C) Correlation of gene copy numbers and gene expression values for 62 squamous cell cancer cases.
(XLSX)

S4 Table Genomic location of “correlation hotspots” of highly correlating genes. (A) Correlation hotspots” from the total NSCLC (n = 190). (B) Correlation hotspots” from the adenocarcinoma cases (n = 105). (C) Correlation hotspots” from the squamous cell cancer cases (n = 62).
(XLSX)

S5 Table Gene Ontology (GO) enrichment analysis. (A) Gene Ontology (GO) enrichment analysis of the 440 significant highly correlating probe sets from the total NSCLC cohort. (B) Gene Ontology (GO) enrichment analysis of the 383 significant highly correlating probe set from the adenocarcinoma cases (n = 105). (C) Gene Ontology (GO) enrichment analysis of the 626 significant highly correlating probe set from the squamous cell cancer cases (n = 62).
(XLSX)

S6 Table Selection of cancer related genes. (A) Genes were selected based on Cancer Gene Census (Futreal et al., 2004). (B) Genes were selected based on described gene copy number gain in previous studies.
(XLSX)

S7 Table Analysis whether the expression of highly correlating genes is associated with survival in the Uppsala cohort. (A) Cox regression model for gene copy number and gene expression in the total NSCLC cohort (n = 190) for the 440 high correlation probe sets. (B) Cox regression model for gene copy number and gene expression in the total NSCLC cohort (n = 105) for the 383 high correlation probe sets. (C) Cox regression model for gene copy number and gene expression in the total NSCLC cohort (n = 62) for the 626 high correlation probe sets.
(XLSX)

S8 Table Meta-analysis of the impact of highly correlating genes on survival using independent NSCLC cohorts. (A) Meta-analysis of the 440 significant highly correlated genes (copy number dependent genes) from the total NSCLC cohort (n = 190). (B) Meta-analysis of the 383 significant highly correlated genes (copy number dependent genes) from the adenocarcinoma cases (n = 105). (C) Meta-analysis of the 626 significant highly correlated genes (copy number dependent genes) from the squamous cell cancer cases (n = 62).
(XLSX)

S9 Table Analysis of highly correlating genes from the Uppsala data set in the TCGA data sets based on Spearman. (A) CNV and gene expression data from 520 adenocarcinoma and 504 squamous cancer cases provided by the TCGA were retrieved from cBioPortal (http://www.cbioportal.org, Gao et al. Sci. Signal. 2013 & Cerami et al. Cancer Discov. 2012). (B) CNV and gene expression data from 520 adenocarcinoma cases provided by the TCGA were retrieved from cBioPortal (http://www.cbioportal.org, Gao et al. Sci. Signal. 2013 & Cerami et al. Cancer Discov. 2012). (C) CNV and gene expression data from 504 squamous cell cancer cases provided by the TCGA were retrieved from cBioPortal (http://www.cbioportal.org, Gao et al. Sci. Signal. 2013 & Cerami et al. Cancer Discov. 2012).
(XLSX)

S10 Table Survival analysis of highly correlating genes in the TCGA data sets. (A) The table lists the 70 highly correlating probe sets that are associated with survival when all NSCLC samples (n = 190) were analysed. The last column showed the unadjusted p-value of the survival analysis based on TCGA data retrieved from the human protein atlas (www.proteinatlas.org, Uhlen et al., 2017, Science). The p-value is based on the log-rank test with an optimised cut-off for each gene. It should be noted that the TCGA data set is not a consecutive cohort like the Uppsala cohort (including large cell carcinoma NOS) and in this analysis 500 adenocarcinomas and 494 squamous cell cancers were just combined. (B) The table lists the 21 highly correlating probe sets that were associated with survival when only adenocarcinoma samples (n = 104) were analysed. The last column showed the unadjusted p-value of the survival analysis based on TCGA data retrieved from the human protein atlas (www.proteinatlas.org, Uhlen et al., 2017, Science). The p-value is based on the log-rank test with an optimised cut-off for each gene for 500 adenocarcinomas cases.
(XLSX)
