# Supplementary figures and images for: Integrative analysis of genome-wide gene copy number changes and gene expression in non-small cell lung cancer
Source: PLoS One. 2017 Nov 7;12(11):e0187246. doi: 10.1371/journal.pone.0187246 (PMC5675410; doi:10.1371/journal.pone.0187246)

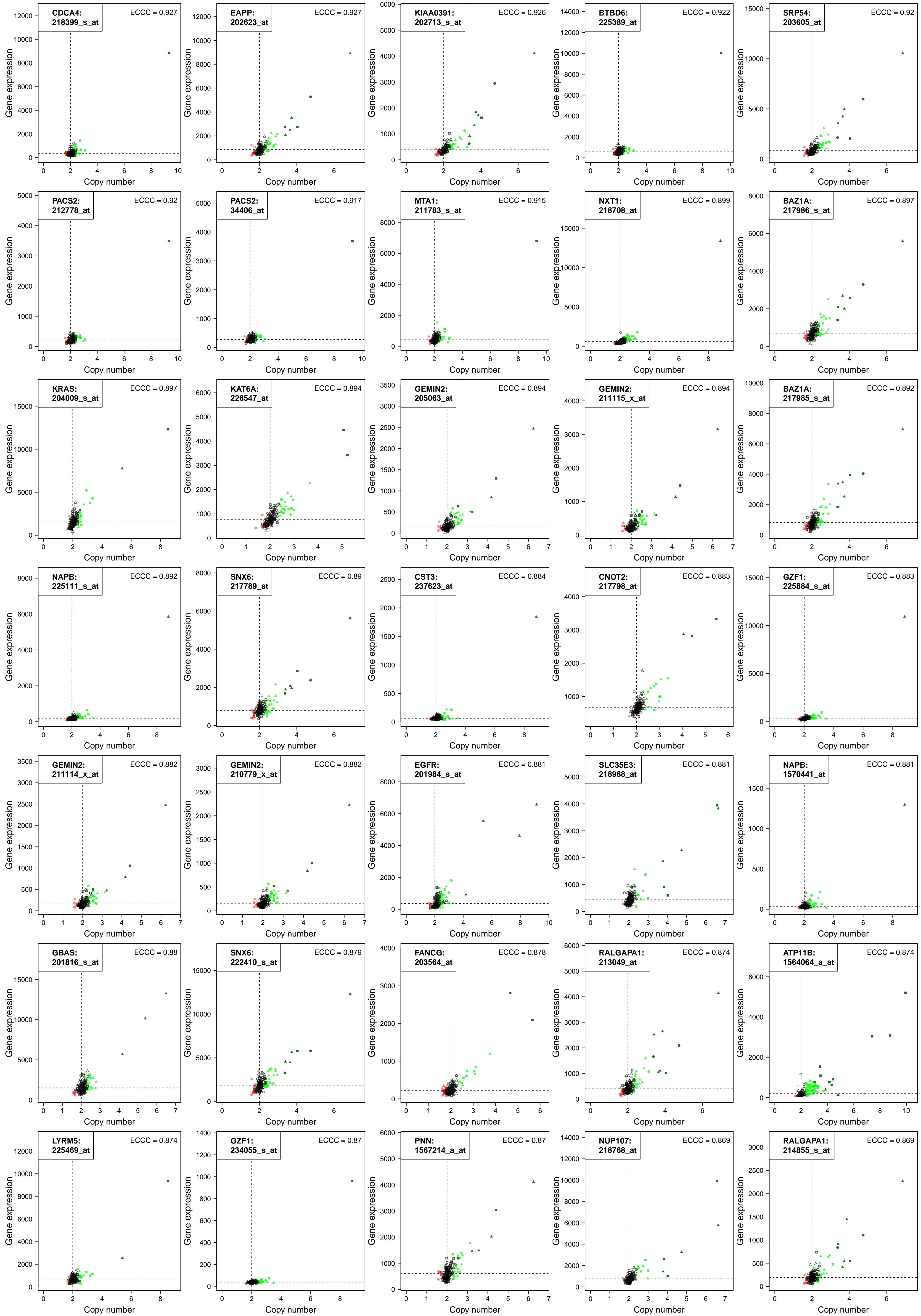

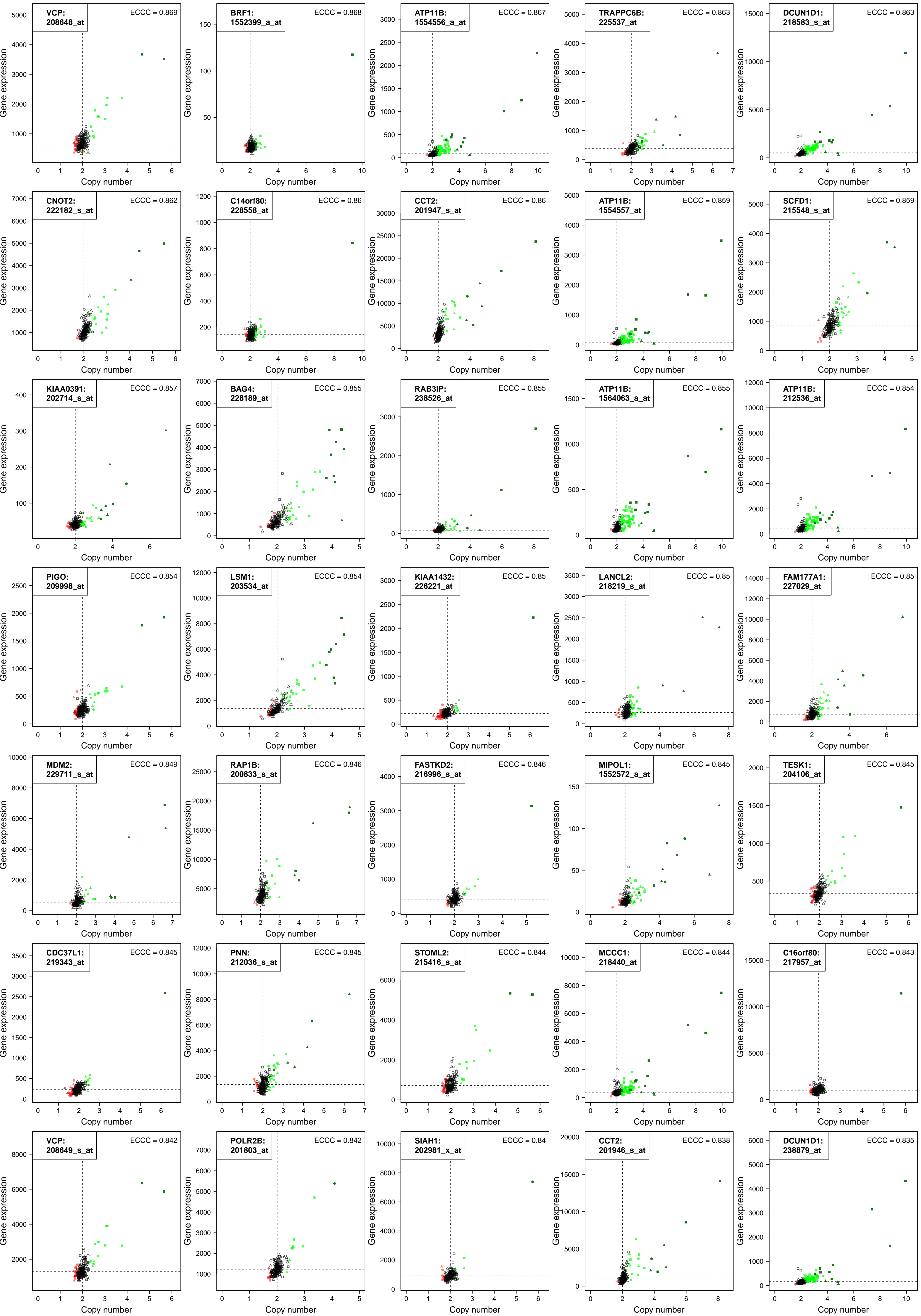

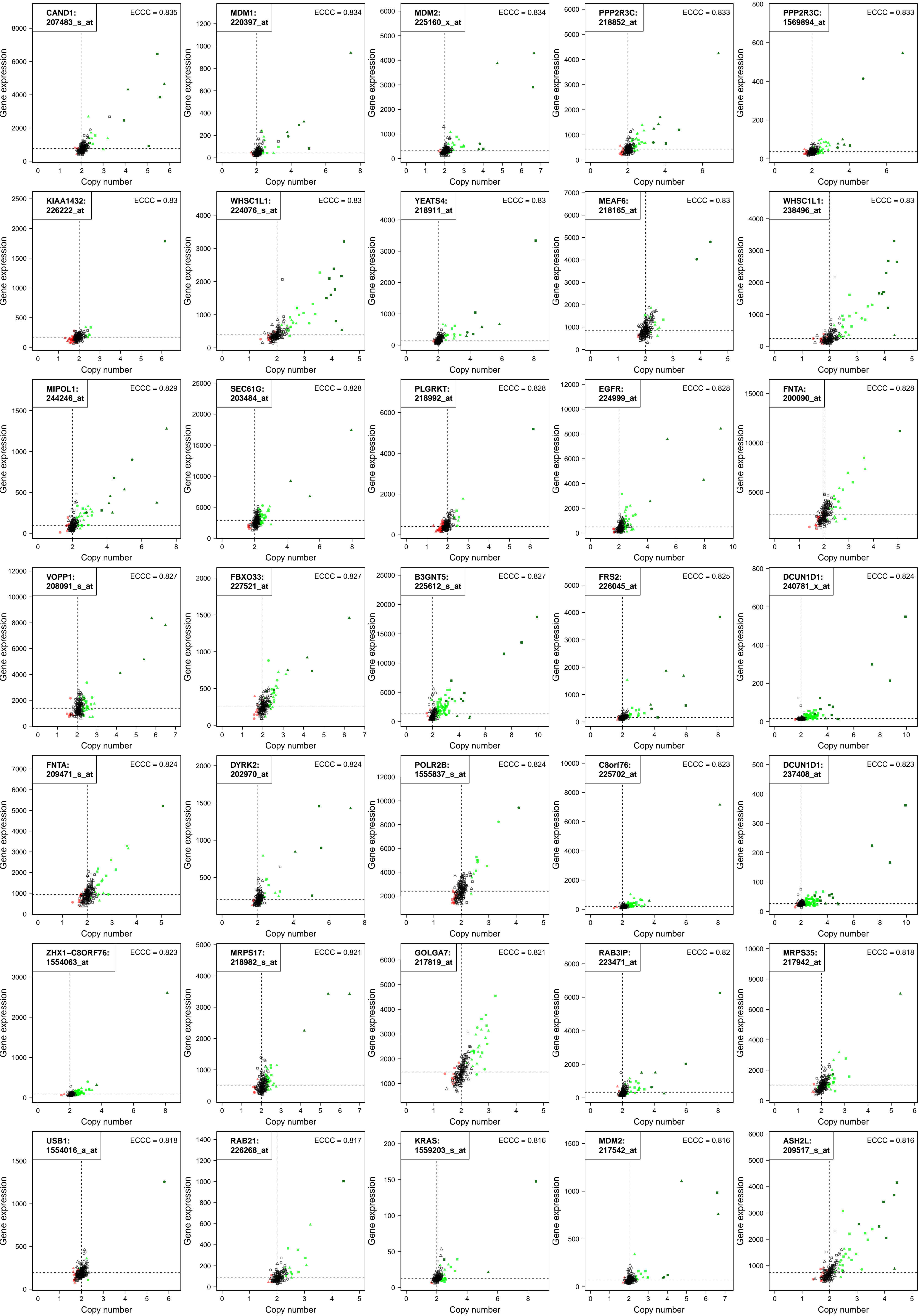

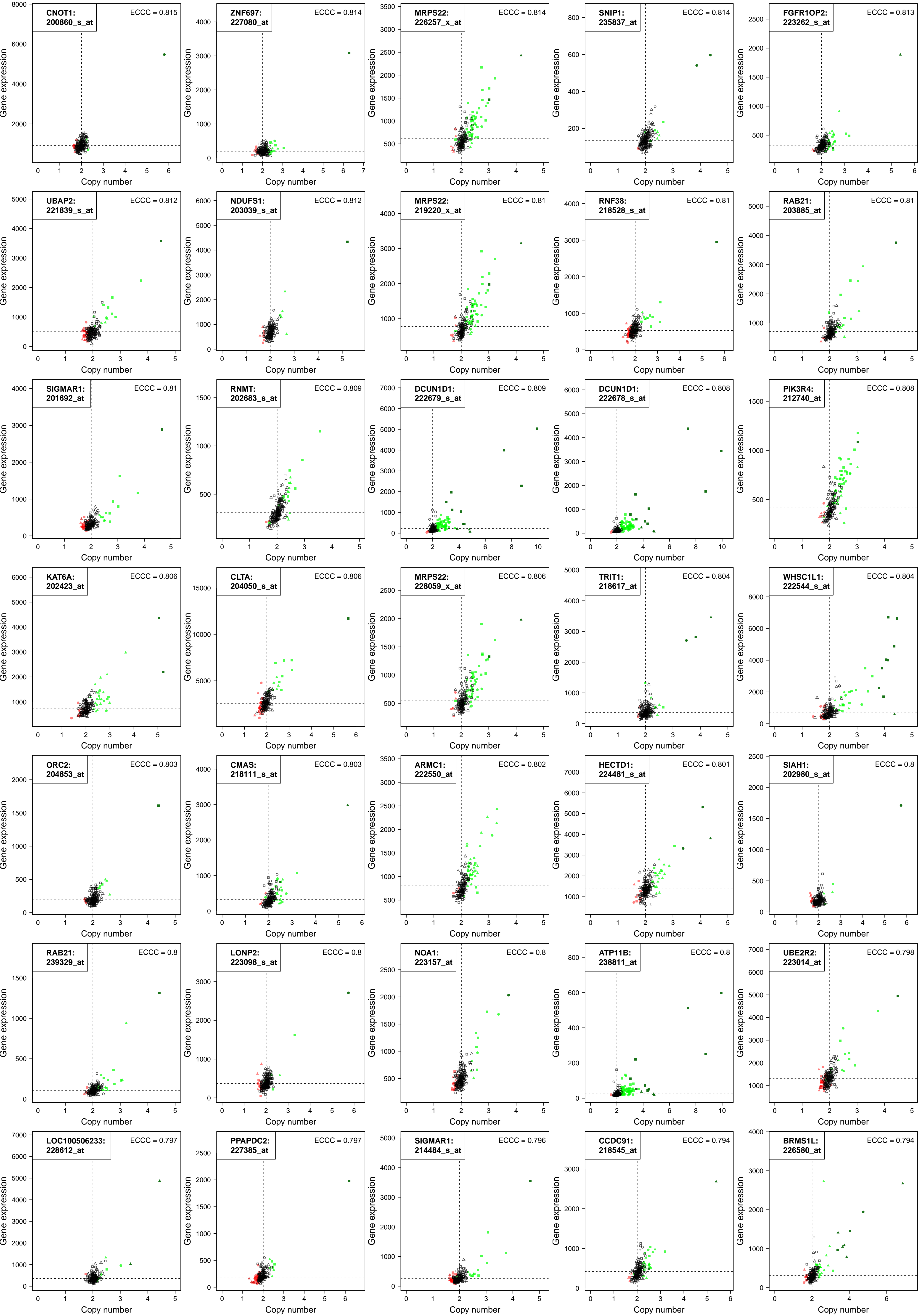

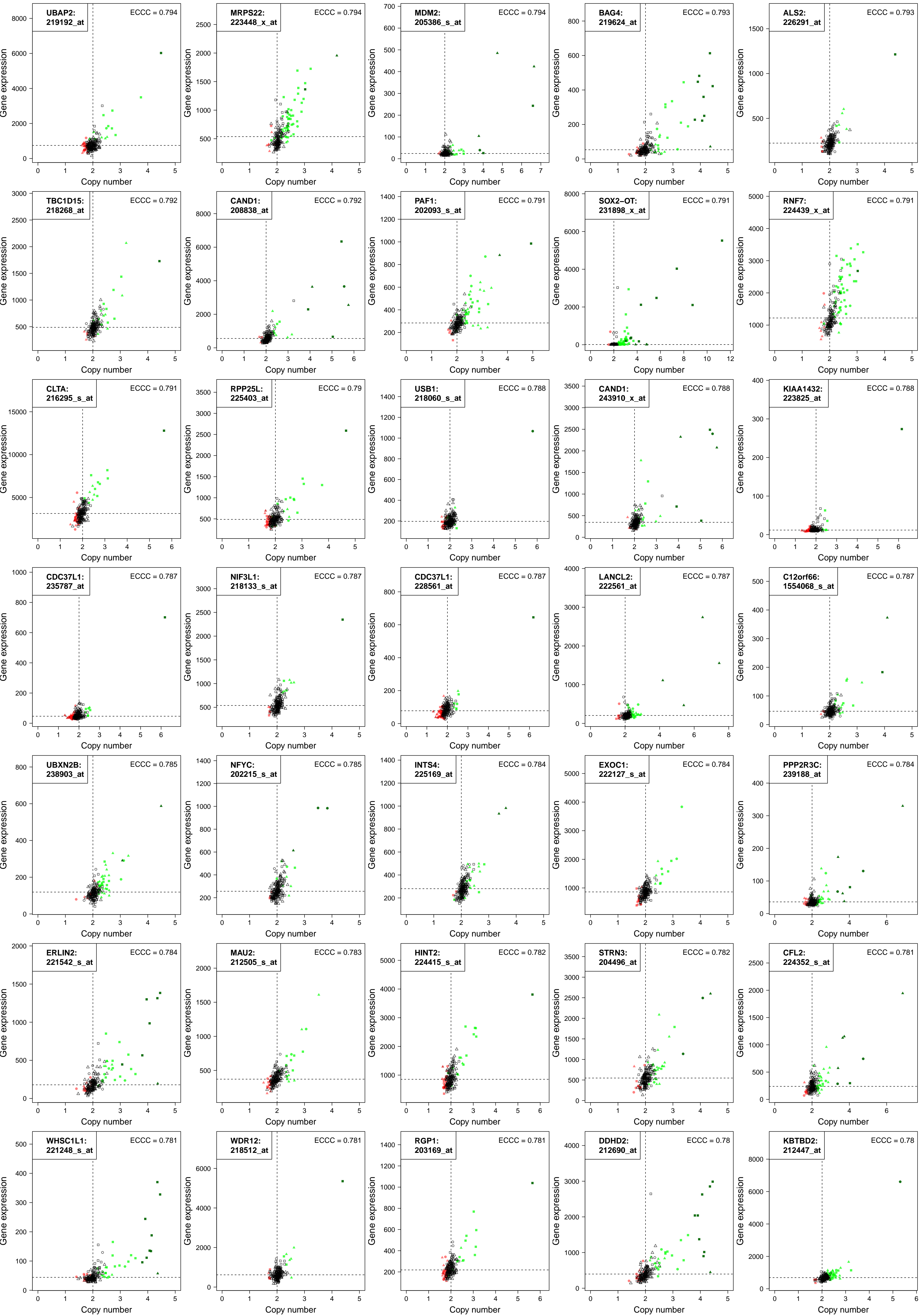

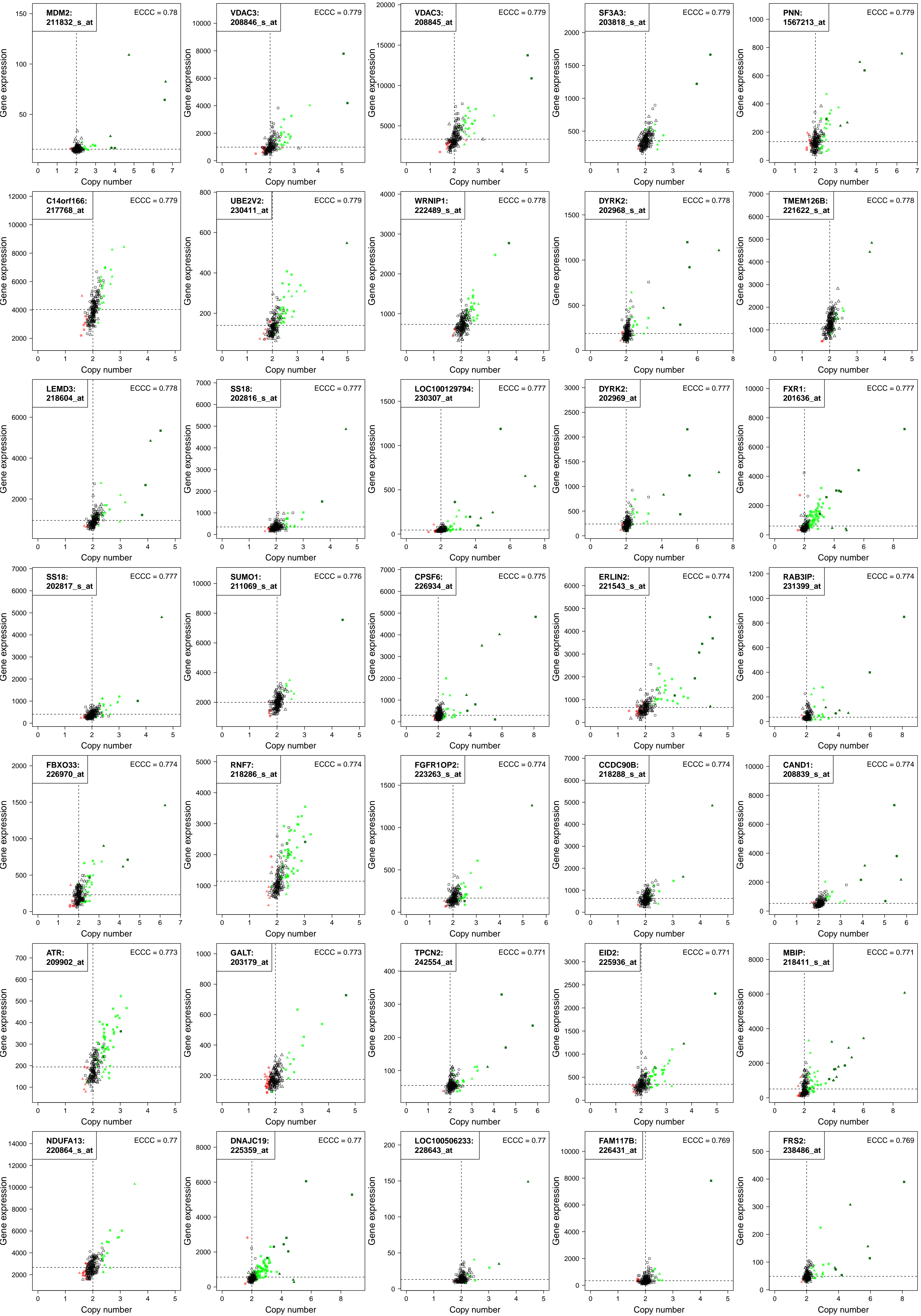

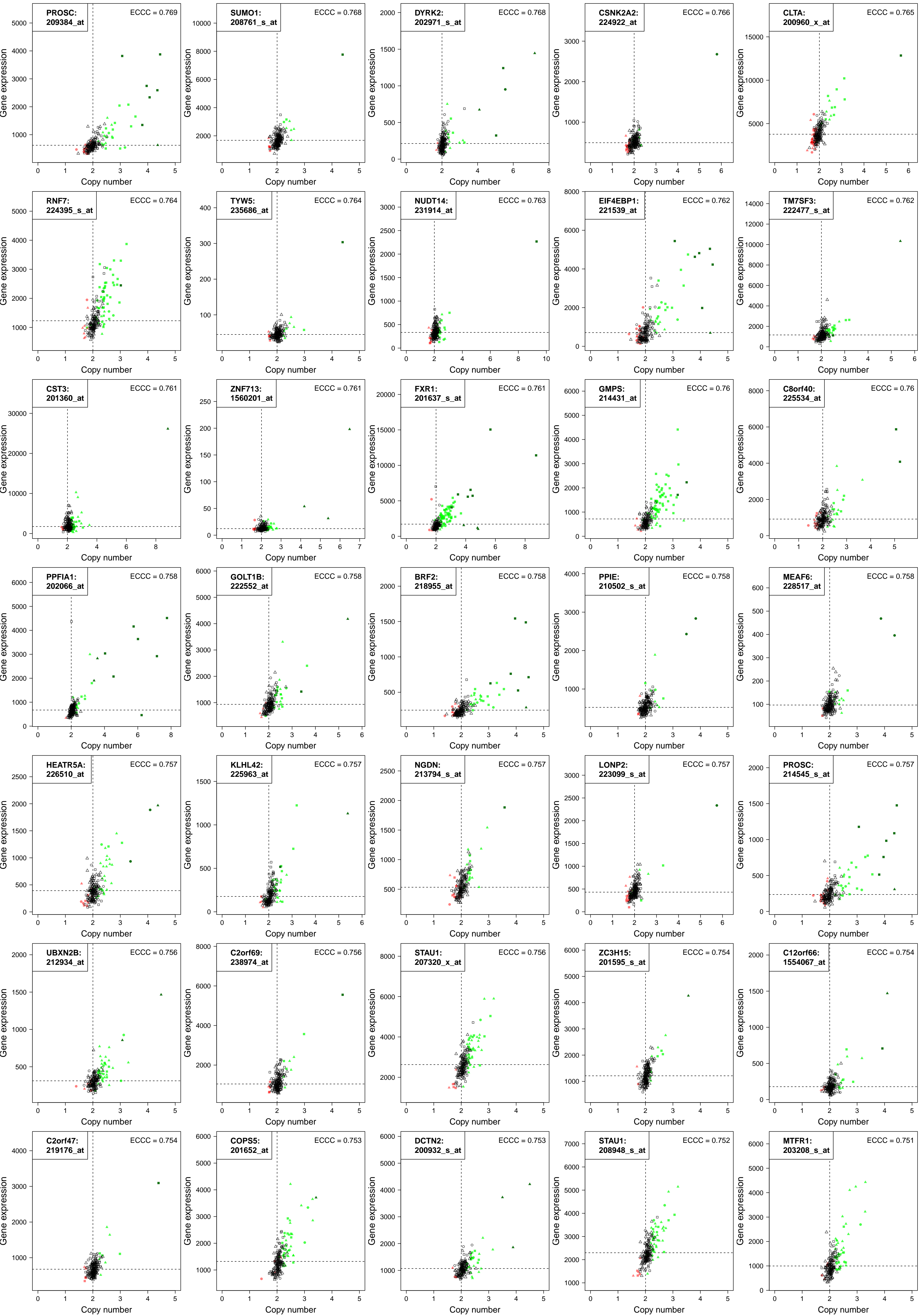

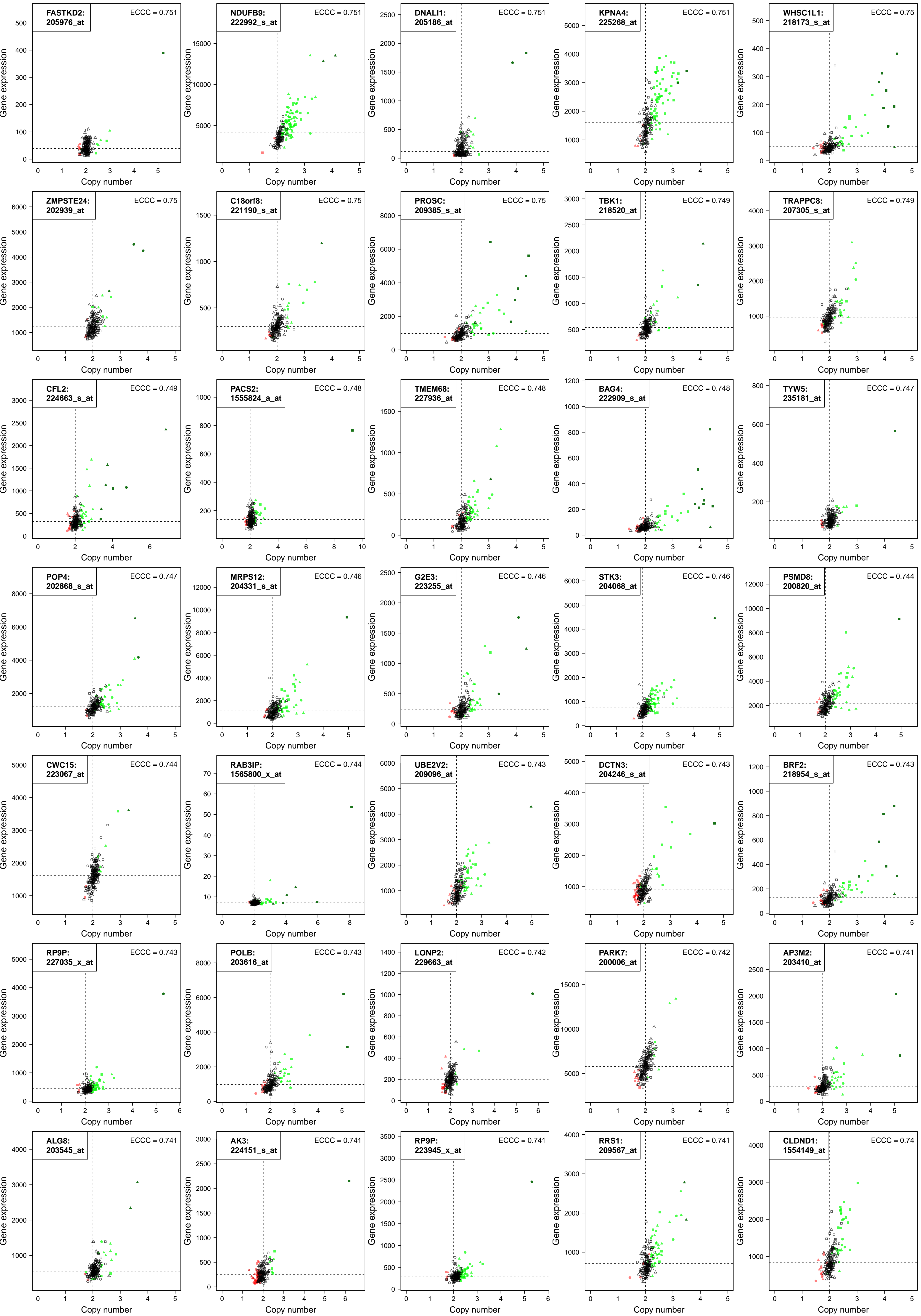

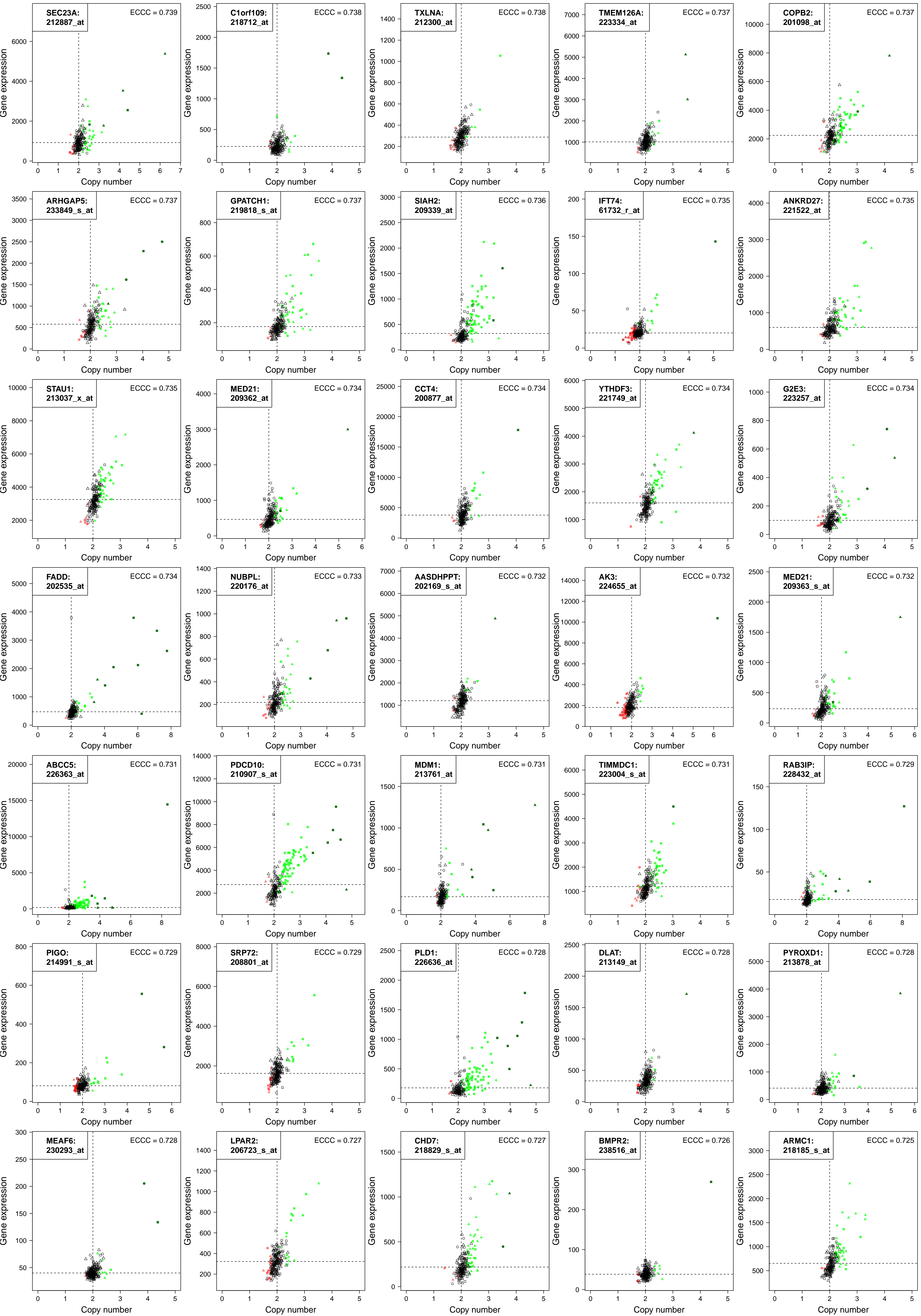

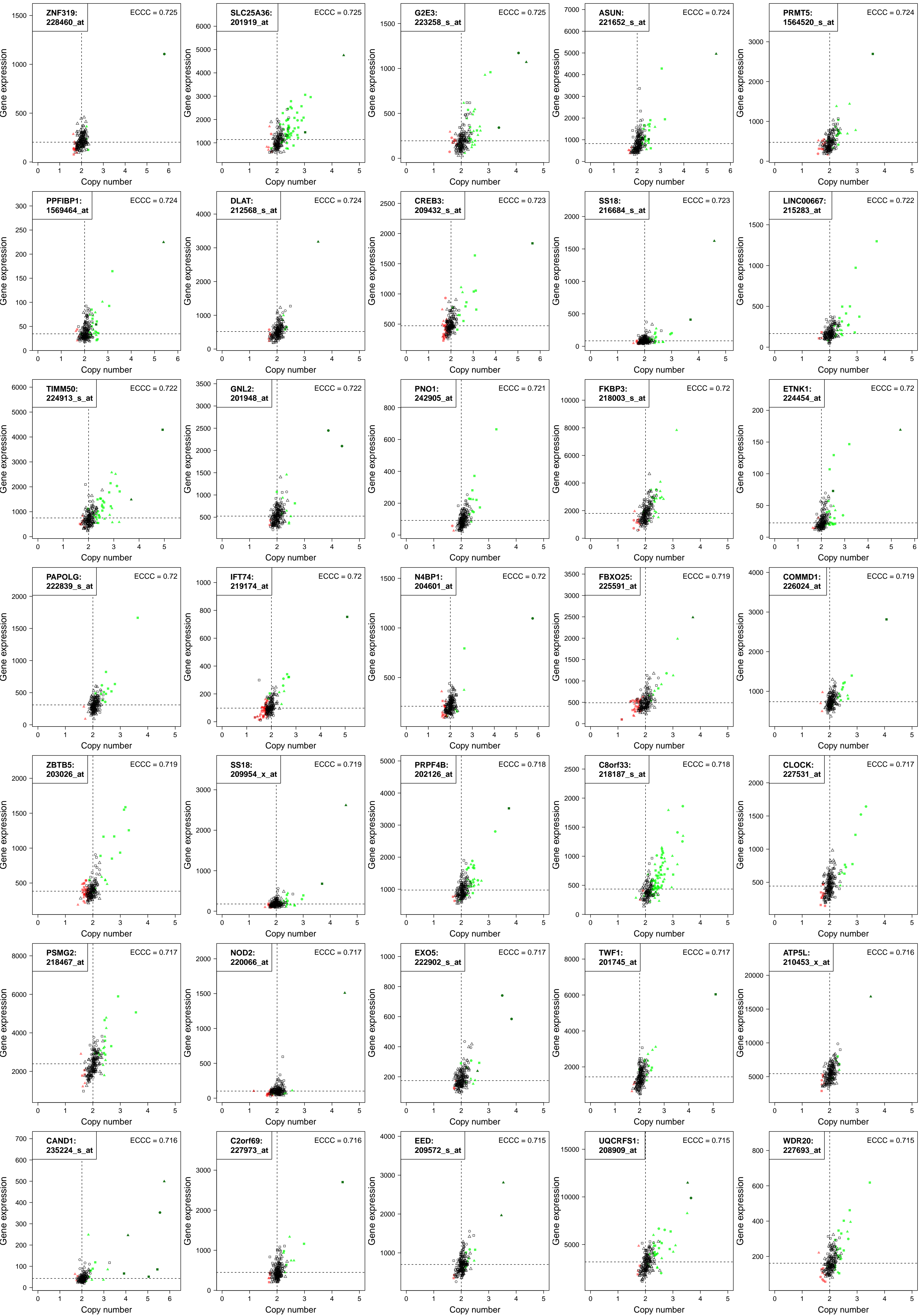

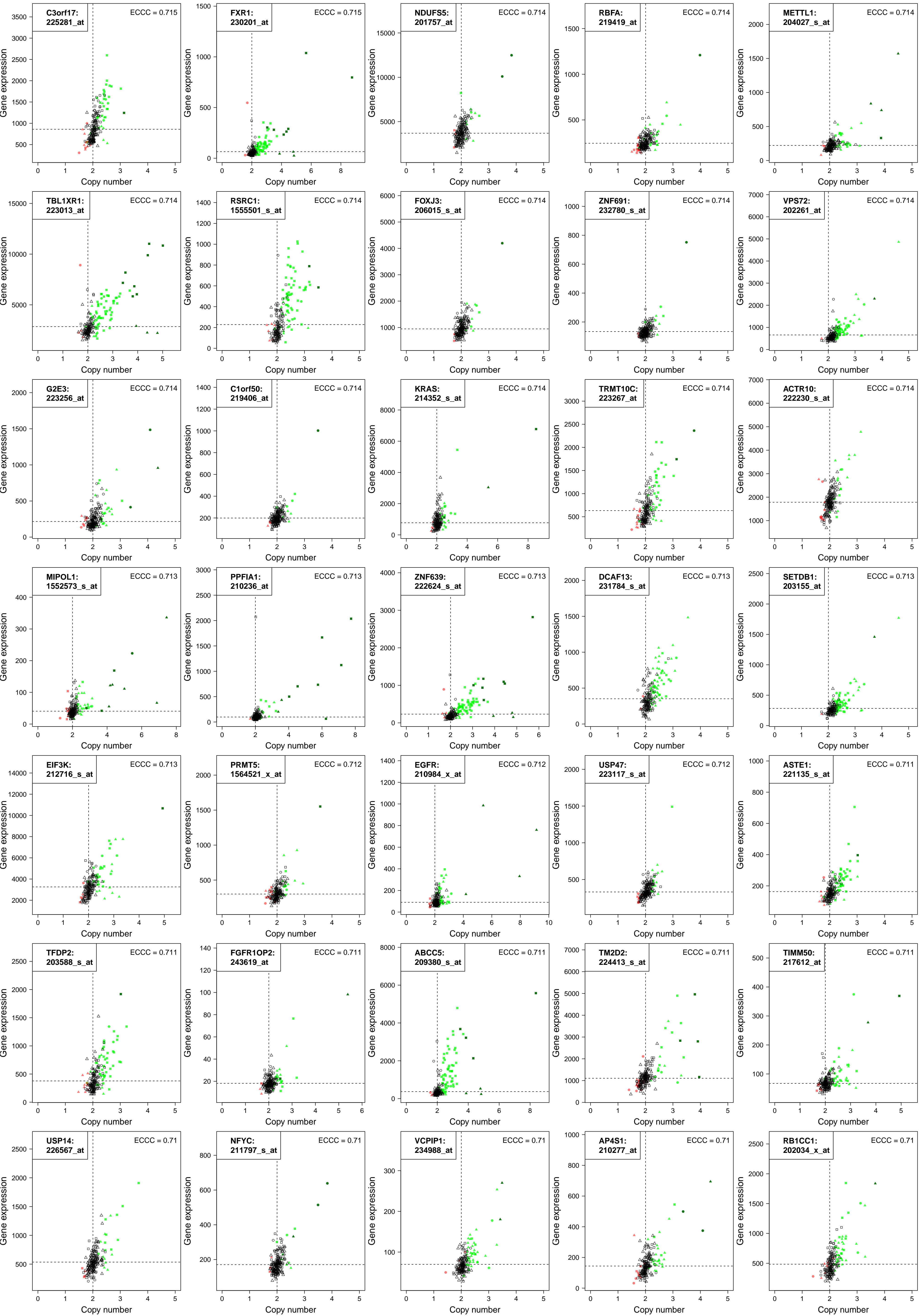

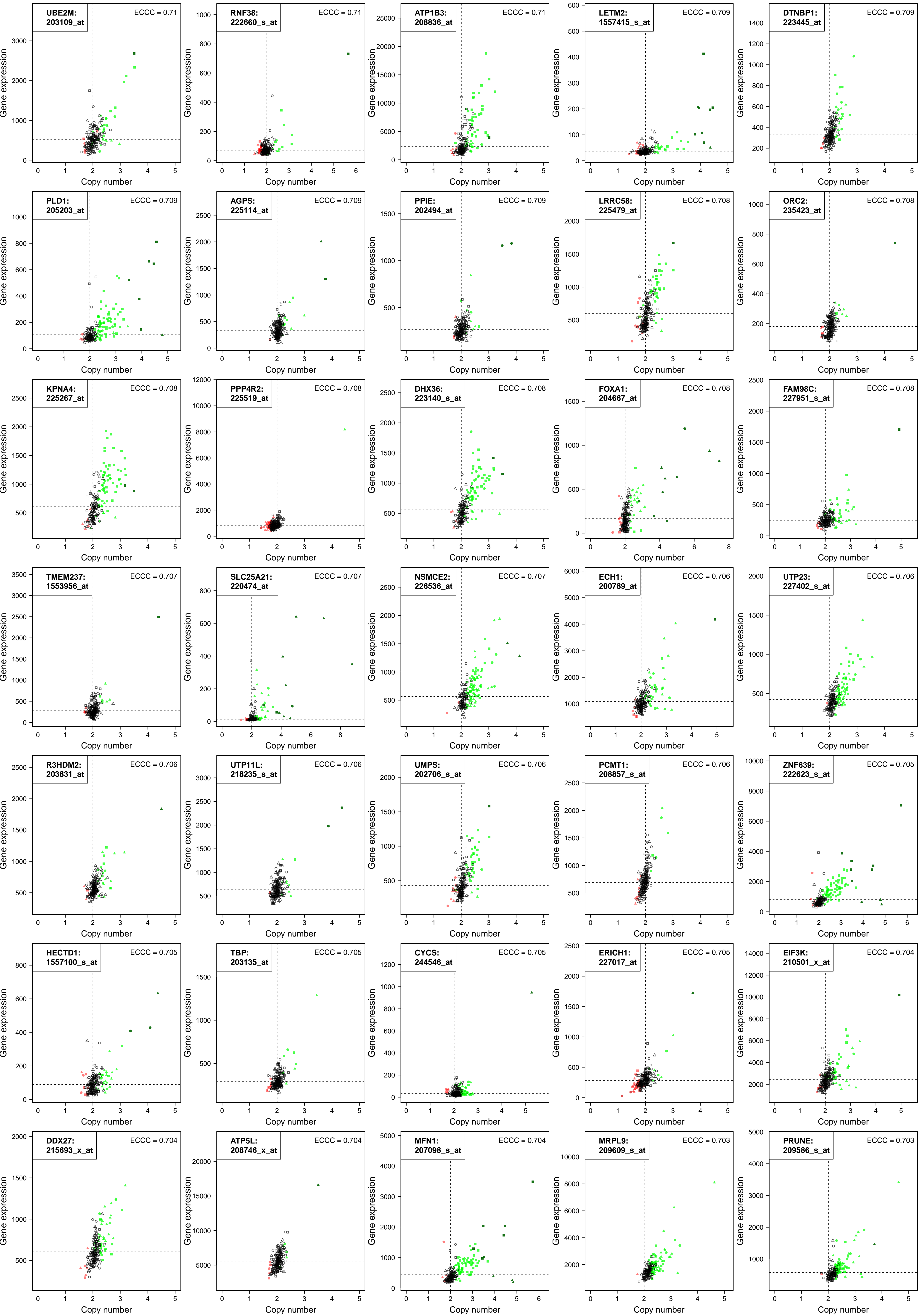

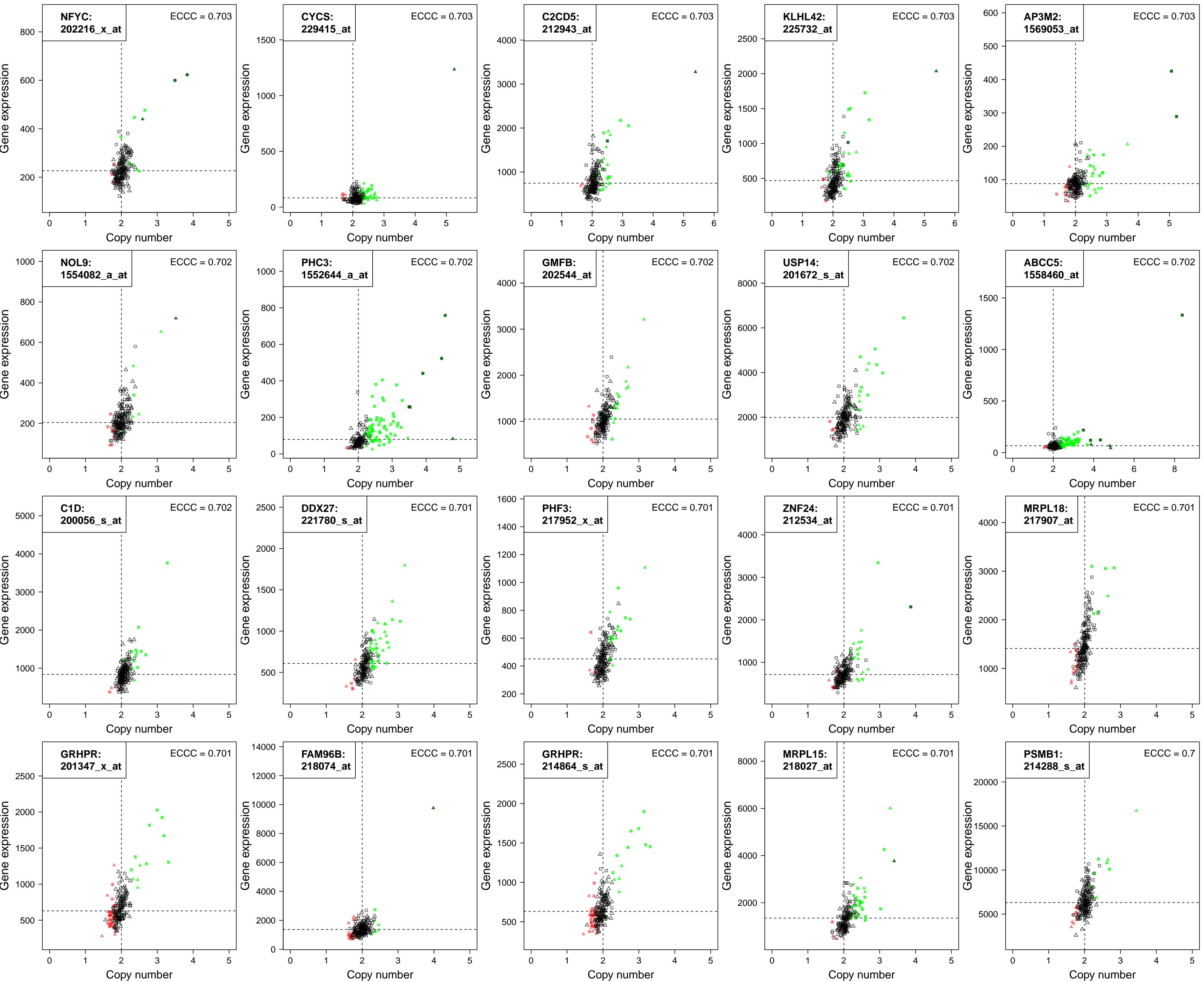

Supplement: S1 Fig — Correlation analysis using the externally centered correlation coefficient (ECCC) revealed 440 probe sets with significant correlations (FDR adj. p < 0.05) and high correlation coefficients (ECCC > 0.7). Gene expression values in a linear scale were plotted against gene copy number values (also linear scale) for all 190 NSCLC cases. Triangular and square symbols represent adenocarcinomas and squamous cell cancer, respectively, while the circular symbols are undifferentiated large cell carcinomas. Dark green represents a high gain, green represents gain and red copy number loss. The correlation is given as externally centered correlation coefficient (ECCC). Abbreviated gene names and corresponding accession numbers of the probe sets are given in the left corner of each plot. (PDF) [file pone.0187246.s001.pdf]

## Slide 1
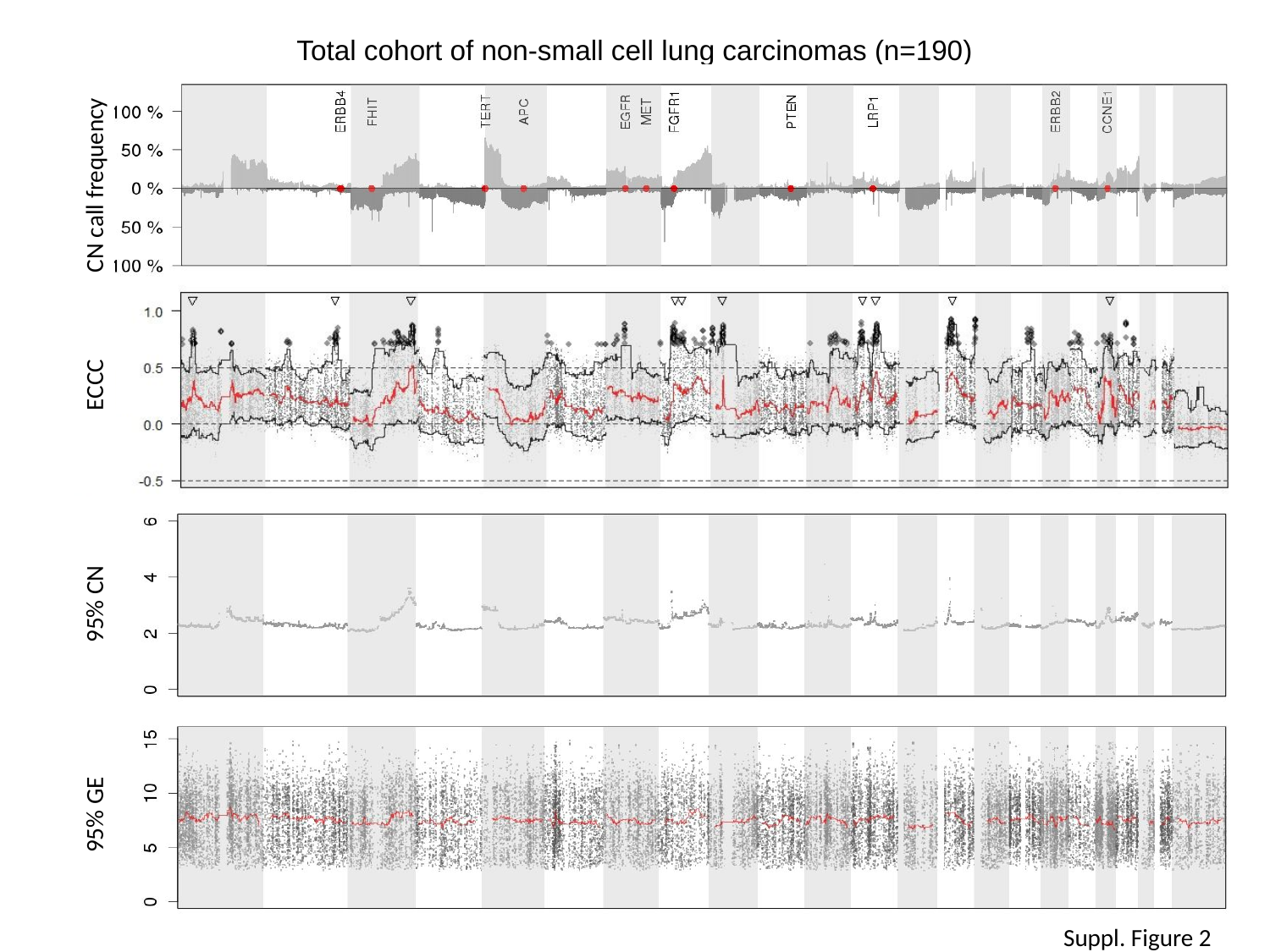

Total cohort of non-small cell lung carcinomas (n=190)
CN call frequency
ECCC
95% CN
95% GE
Suppl. Figure 2

Supplement: S2 Fig — The total cohort with 190 patients is shown. The panels give copy number (CN) call frequencies (from Fig 1), the externally centered correlation coefficients (ECCC) (triangles: “hotspot regions” of high correlation), the 95% quantile of the segmented gene copy number values (95% CN), and the 95% quantile (grey dots) and the moving average (red line) of the logarithmic gene expression values. The eight ECCC hotspot regions on chromosomes 3, 8, 9, 12, 14 and 19 coincide with higher values of the 95% CN. (PPTX) [file pone.0187246.s002.pptx]

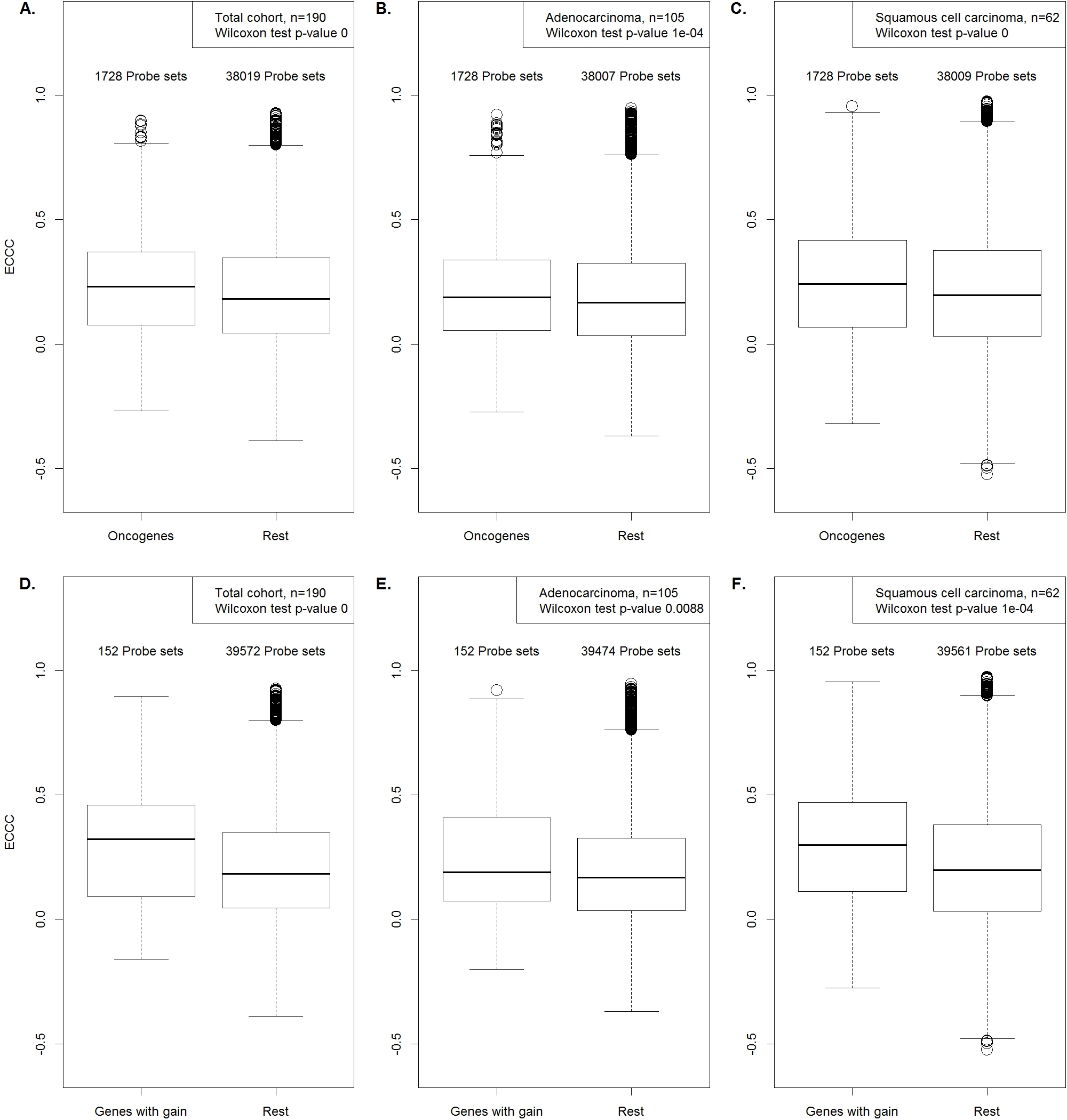

Supplement: S3 Fig — Probe sets for cancer genes (n = 522) were selected based on Cancer Gene Census (Futreal et al. 2004) and the externally centered correlation coefficients (ECCC) were compared to the ECCC of all other probe sets (A-C). In addition, 152 probe sets for 46 genes were listed that previously have been reported to show genomic gain in NSCLC (D-F). (JPG) [file pone.0187246.s003.jpg]

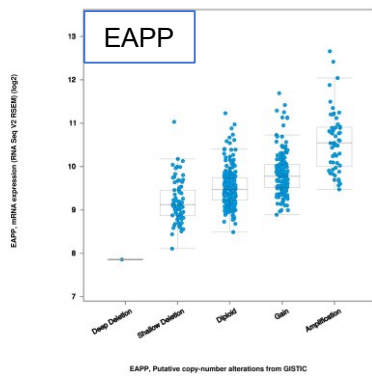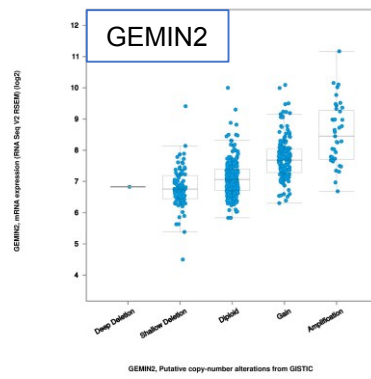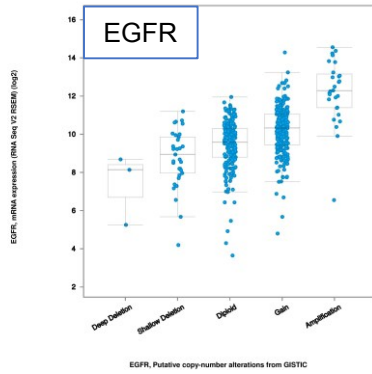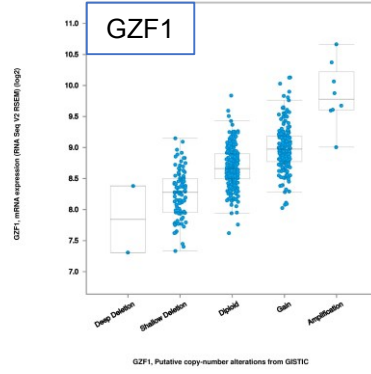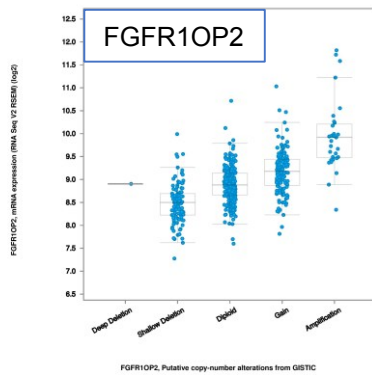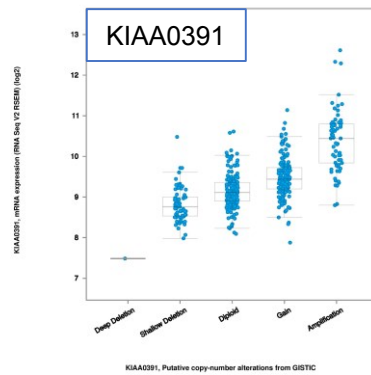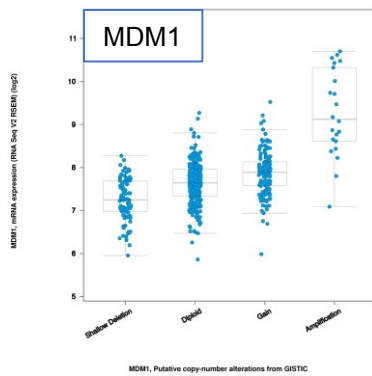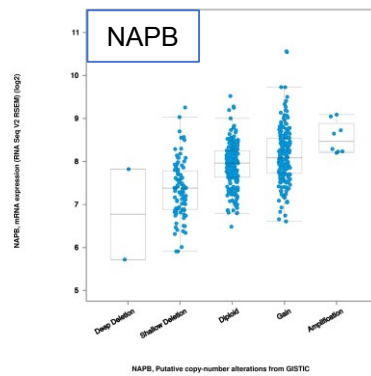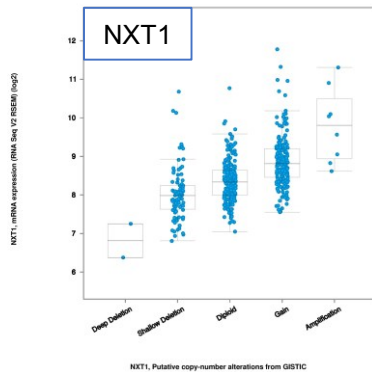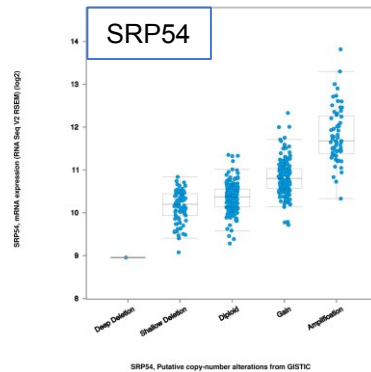

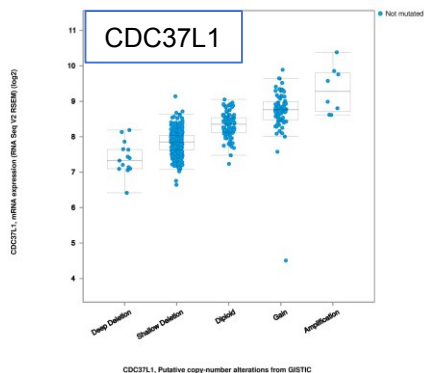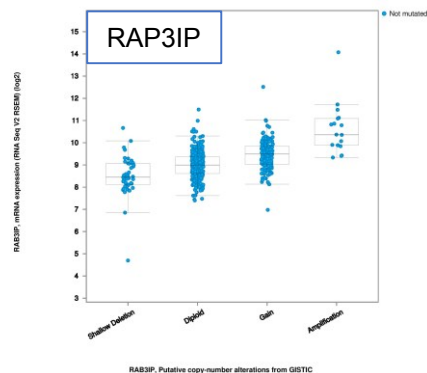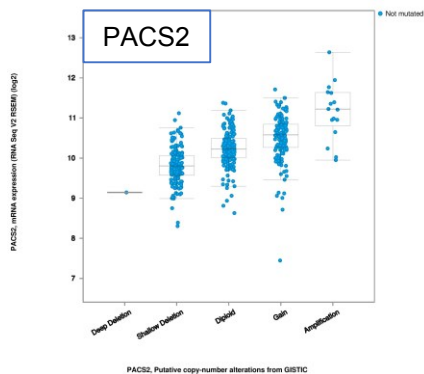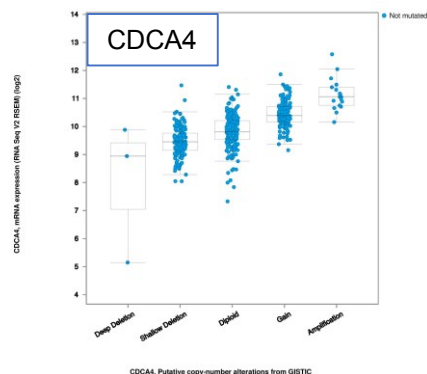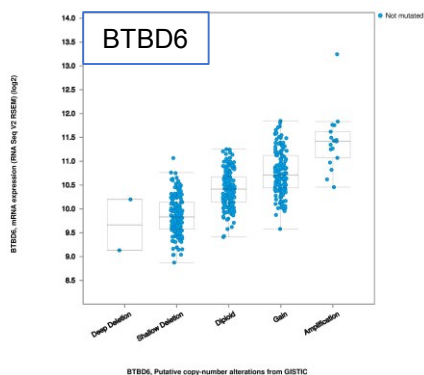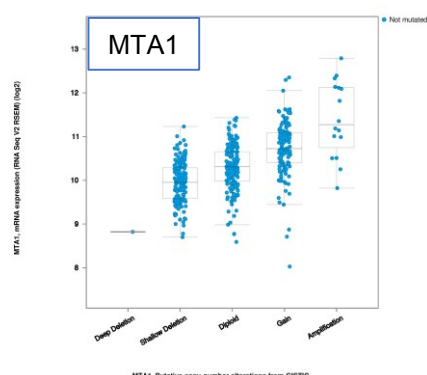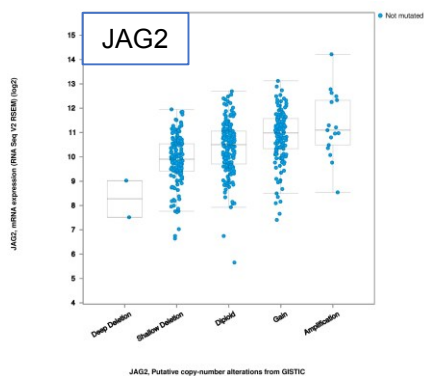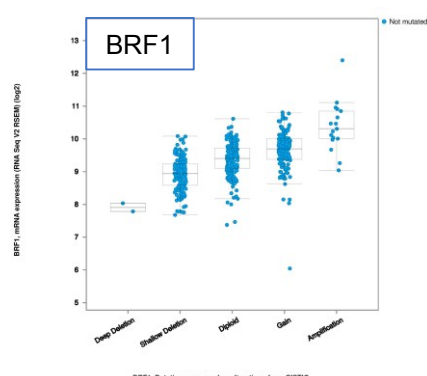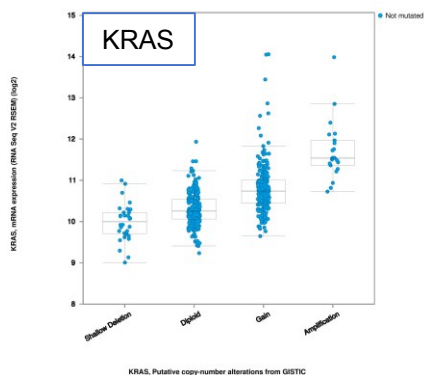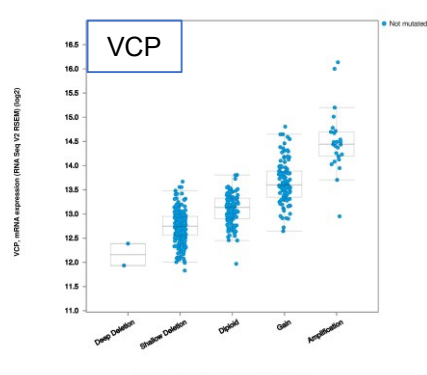

Supplement: S4 Fig — The box plots were based on gene copy numbers (estimated by GISTIC 2.0) and gene expression data from 520 adenocarcinomas and 504 squamous cancer cases provided by the TCGA were downloaded from cBioPortal (August 2017; http://www.cbioportal.org, Gao et al. Sci. Signal. 2013 & Cerami et al. Cancer Discov. 2012). (PDF) [file pone.0187246.s004.pdf]
