# Supplementary material for: Integrative analysis of genome-wide gene copy number changes and gene expression in non-small cell lung cancer
Source: PLoS One. 2017 Nov 7;12(11):e0187246. doi: 10.1371/journal.pone.0187246 (PMC5675410; doi:10.1371/journal.pone.0187246)
Supplement: S1 Table — Patient characteristics of 190 patients with available fresh frozen tissue. Tumor tissue was used for concurrent analysis of genome wide gene copy number and mRNA expression levels. (DOCX) [file pone.0187246.s005.docx]

**S1 Table:** Patient characteristics of 190 patients with available fresh frozen tissue. Tumor tissue was used for concurrent analysis of genome wide gene copy number and mRNA expression levels.

|  | N (%) |
| --- | --- |
| All cases | 190 (100.0) |
| Sex |  |
| Male | 103 (54.2) |
| Female | 87 (45.8) |
| Age at diagnosis |  |
| ≤ 70 | 129 (67.9) |
| > 70 | 61 (32.1) |
| Median (range) | 65 (39-85) |
| Smoking History |  |
| Ever smoker | 175 (92.1) |
| Never smoker | 15 (7.9) |
| Stage at diagnosis |  |
| IA | 37 (19.5) |
| IB | 87 (45.8) |
| IIA | 6 (3.2) |
| IIB | 29 (15.3) |
| IIIA | 21 (11.1) |
| IIIB | 6 (3.2) |
| IV | 4 (2.1) |
| Histology |  |
| Squamous cell carcinoma | 62 (32.6) |
| Adenocarcinoma | 105 (55.3) |
| Large cell carcinoma (NOS) | 23 (12.1) |
| WHO performance status |  |
| 0 | 104 (54.7) |
| 1 | 70 (36.8) |
| 2 | 12 (6.3) |
| 3 | 4 (2.1) |
| Mean follow-up (months) | 59.9 |
