# Supplementary material for: Integrative analysis of genome-wide gene copy number changes and gene expression in non-small cell lung cancer
Source: PLoS One. 2017 Nov 7;12(11):e0187246. doi: 10.1371/journal.pone.0187246 (PMC5675410; doi:10.1371/journal.pone.0187246)
Supplement: S2 Table — The table lists gains or losses observed in the present study (Uppsala) and indicates whether similar aberrations in the same chromosomal region have also been reported in previous studies. (DOCX) [file pone.0187246.s006.docx]

**S2 Table:** Genomic aberrations in the Uppsala study cohort compared to previous results. The table lists gains or losses observed in the present study (Uppsala) and indicates whether similar aberrations in the same chromosomal region have also been reported in previous studies.

1. **Adenocarcinomas**

| Uppsala  (this study) | TCGH  2014 ^19 #^ | Lazar et al., 2013 ^15^ | Thu et al.  2012 ^55^ * | Iwakawa et al., 2011 ^56^ **^§^** | Lu et al., 2011 ^57^ | Weir et al., 2007 ^58^ |
| --- | --- | --- | --- | --- | --- | --- |
| Affy 250 K | Affymetrix 6.0 | Agilent CGH  7500 | Affymetrix 6.0 | Affymetrix 250K | Affymetrix 6.0 | Affymetrix  250K |
| n=105 | n=230 | n=50 | n=39* | n=60 | n=42 | N=371 |
| Gain |  |  |  |  |  |  |
| 1p  1q | 1p  1q | 1q | 1q |  | 1q | 1q |
| 2q |  |  |  |  |  |  |
|  | 3p  3q |  |  |  |  |  |
| 5p | 5p  5q | 5p | 5p | 5p | 5p  5q | 5p |
|  | 6p |  | 6p |  |  | 6p |
| 7p | 7p  7q |  |  | 7p | 7p | 7p  7q |
| 8q | 8p  8q | 8q | 8q | 8q | 8q | 8q |
| 11q | 11q |  |  |  |  |  |
|  | 12p  12q |  |  |  |  |  |
| 14q | 14q |  |  | 14q | 14p  14q |  |
| 16q |  |  |  |  |  | 16p |
|  | 17q | 17q | 17q |  |  | 17q |
|  | 18q |  |  |  |  |  |
|  | 19q |  |  |  |  |  |
| 20p  20q | 20p  20q | 20q | 20q |  |  | 20p  20q |
|  | Xq |  |  |  |  |  |
| Loss |  |  |  |  |  |  |
|  | 1p | 1p |  |  | 1p |  |
|  | 2q |  |  |  |  |  |
|  | 3p  3q | 3p |  |  | 3p | 3p |
|  | 4p  4q | 4q |  |  |  |  |
|  | 5p  5q | 5q |  |  |  | 5q |
|  | 6q | 6q | 6q |  | 6q | 6q |
|  | 8p | 8p |  |  | 8p |  |
| 9p | 9p | 9p  9q | 9p |  | 9p  9q | 9p  9q |
|  | 10p  10q |  |  |  | 10p  10q | 10q |
|  | 11q |  |  |  |  |  |
|  | 12p  12q |  |  |  | 12p | 12p |
|  | 13q | 13p  13q |  |  | 13p | 13q |
|  | 14q |  |  |  |  |  |
|  | 15q | 15p | 15p |  |  | 15q |
|  | 16q |  |  |  |  |  |
|  | 17p | 17p |  |  | 17p | 17p |
|  | 18q |  | 18q |  | 18p  18q | 18q |
|  | 19p  19q |  |  |  | 19p  19q | 19p  19q |
|  | 21q |  |  |  |  | 21q |
|  | 22q |  |  |  |  | 22q |
|  | Xp |  |  |  |  |  |

^#^ arms with focal aberrations identified by GISTIC; *only smokers; **^§^** small sized adenocarcinoma (< 2cm), only gains were given.

**B) Squamous cell cancer**

| Uppsala  (this study) | Lo et al., 2008 ^14^ | Lazar et al., 2013 ^15^ | Weiss et al., 2010 ^&^ | TCGA, 2012 ^18^ |
| --- | --- | --- | --- | --- |
| Affy 250 K | BAC array | Agilent 7500 | Affymetrix 6.0 | Agilent 1M |
| n=64 | n=12 | n=46 | n=155 | n=122 |
| Gain |  |  |  |  |
| 1p  1q |  | 1q | 1p | 1q |
| 2q |  | 2p | 2p  2q | 2p |
| 3q | 3q | 3q | 3q | 3q |
|  |  |  | 4q |  |
| 5p | 5p | 5p | 5p | 5p |
|  |  |  | 6p |  |
|  |  |  | 7p | 7p  7q |
| 8p  8q |  |  | 8p  8q | 8q |
|  |  |  | 9p |  |
| 11q |  |  | 11q |  |
| 12p |  |  | 12p  12q | 12p |
| 14q |  |  | 14q |  |
| 15q |  |  |  |  |
| 16q |  |  |  |  |
| 17q |  |  |  |  |
|  | 18p  18q |  | 18q |  |
| 19q |  |  | 19q |  |
| 20p  20q | 20p  20q | 20p  20q | 20p |  |
|  |  |  | 22q |  |
| Loss |  |  |  |  |
| 1p | 1p | 1p  1q | 1p  1q | 1p |
|  |  |  | 2q |  |
| 3p | 3p | 3p | 3p  3q | 3p |
| 4q | 4p | 4p  4q | 4p  4q | 4p  4q |
| 5q |  |  |  | 5q |
|  | 7  (telomeric region) |  | 7p  7q | 7p  7q |
| 8p |  | 8p | 8q | 8p |
| 9p | 9p | 9p |  | 9p  9q |
|  | 10q (distal tip) | 10p  10q | 10p | 10q |
| 13q |  | 13p  13q | 10q | 13p  13q |
|  |  |  | 14q |  |
| 15q |  |  |  |  |
|  |  | 16p  16q | 16p |  |
| 17p | 17p  17q | 17p | 17p | 17p |
|  |  | 18q | 18q | 18q |
|  | 19p  19q |  |  | 19p |
|  | 21q | 21p  21q |  | 21p  21q |
| 22q | 22p  22q |  |  |  |
| Xp |  |  |  |  |

^&^ arms with focal aberrations identified by GISTIC

**References**

Cancer Genome Atlas Research Network. Comprehensive molecular profiling of lung adenocarcinoma. Nature. 2014 511:543-50.

Cancer Genome Atlas Research Network. Comprehensive genomic characterization of squamous cell lung cancers. Nature. 2012 489:519-25.

Iwakawa R, Kohno T, Kato M, Shiraishi K, Tsuta K, Noguchi M, Ogawa S, Yokota, J. MYC amplification as a prognostic marker of early-stage lung adenocarcinoma identified by whole genome copy number analysis. Clin Cancer Res. 2011 17:1481-9.

Lazar V, Suo C, Orear C, van den Oord J, Balogh Z, Guegan J, Job B, Meurice G, Ripoche H, Calza S, Hasmats J, Lundeberg J, Lacroix L, Vielh P, Dufour F, Lehtiö J, Napieralski R, Eggermont A, Schmitt M, Cadranel J, Besse B, Girard P, Blackhall F, Validire P, Soria JC, Dessen P, Hansson J, Pawitan Y. Integrated molecular portrait of non-small cell lung cancers. BMC Med Genomics. 2013 6:53.

Lo KC, Stein LC, Panzarella JA, Cowell JK, Hawthorn L. Identification of genes involved in squamous cell carcinoma of the lung using synchronized data from DNA copy number and transcript expression profiling analysis. Lung Cancer. 2008 59:315-31.

Lu TP, Lai LC, Tsai MH, Chen PC, Hsu CP, Lee JM, Hsiao CK, Chuang EY. Integrated analyses of copy number variations and gene expression in lung adenocarcinoma. PLoS One. 2011 6:e24829.

Thu KL, Vucic EA, Chari R, Zhang W, Lockwood WW, English JC, Fu R, Wang P, Feng Z, MacAulay CE, Gazdar AF, Lam S, Lam WL. Lung adenocarcinoma of never smokers and smokers harbor differential regions of genetic alteration and exhibit different levels of genomic instability. PLoS One. 2012 7:e33003.

Weir BA, Woo MS, Getz G, Perner S, Ding L, Beroukhim R, Lin WM, Province MA, Kraja A, Johnson LA, Shah K, Sato M, Thomas RK, Barletta JA, Borecki IB, Broderick S, Chang AC, Chiang DY, Chirieac LR, Cho J, Fujii Y, Gazdar AF, Giordano T, Greulich H, Hanna M, Johnson BE, Kris MG, Lash A, Lin L, Lindeman N, Mardis ER, McPherson JD, Minna JD, Morgan MB, Nadel M, Orringer MB, Osborne JR, Ozenberger B, Ramos AH, Robinson J, Roth JA, Rusch V, Sasaki H, Shepherd F, Sougnez C, Spitz MR, Tsao MS, Twomey D, Verhaak RG, Weinstock GM, Wheeler DA, Winckler W, Yoshizawa A, Yu S, Zakowski MF, Zhang Q, Beer DG, Wistuba II, Watson, MA, Garraway LA, Ladanyi M, Travis WD, Pao W, Rubin MA, Gabriel SB, Gibbs RA, Varmus HE, Wilson RK, Lander ES, Meyerson M. Characterizing the cancer genome in lung adenocarcinoma. Nature. 2007 450:893-8.

Weiss J, Sos ML, Seidel D, Peifer M, Zander T, Heuckmann JM, Ullrich RT, Menon R, Maier S, Soltermann A, Moch H, Wagener P, Fischer F, Heynck S, Koker M, Schöttle J, Leenders F, Gabler F, Dabow I, Querings S, Heukamp LC, Balke-Want H, Ansén S, Rauh D, Baessmann I, Altmüller J, Wainer Z, Conron M, Wright G, Russell P, Solomon B, Brambilla E, Brambilla C, Lorimier P, Sollberg S, Brustugun OT, Engel-Riedel W, Ludwig C, Petersen I, Sänger J, Clement J, Groen H, Timens W, Sietsma H, Thunnissen E, Smit E, Heideman D, Cappuzzo F, Ligorio C, Damiani S, Hallek M, Beroukhim R, Pao W, Klebl B, Baumann M, Buettner R, Ernestus K, Stoelben E, Wolf J, Nürnberg P, Perner S, Thomas RK. Frequent and focal FGFR1 amplification associates with therapeutically tractable FGFR1 dependency in squamous cell lung cancer. Sci Transl Med. 2010 15:62ra93.
